# Different Patterns of Neurodegeneration and Glia Activation in CA1 and CA3 Hippocampal Regions of TgCRND8 Mice

**DOI:** 10.3389/fnagi.2018.00372

**Published:** 2018-11-13

**Authors:** Filippo Ugolini, Daniele Lana, Pamela Nardiello, Daniele Nosi, Daniela Pantano, Fiorella Casamenti, Maria Grazia Giovannini

**Affiliations:** ^1^Department of Health Sciences, Section of Clinical Pharmacology and Oncology, University of Florence, Florence, Italy; ^2^Department of Neuroscience, Psychology, Drug Research and Child Health, NEUROFARBA, Section of Pharmacology and Toxicology, University of Florence, Florence, Italy; ^3^Department of Experimental and Clinical Medicine, University of Florence, Florence, Italy

**Keywords:** Alzheimer’s disease, microglia, astrocytes, TNF-α, IL1β, iNOS, apoptosis, confocal immunohistochemistry

## Abstract

We investigated the different patterns of neurodegeneration and glia activation in CA1 and CA3 hippocampal areas of TgCRND8 mice. The main feature of this transgenic model is the rapid development of the amyloid pathology, which starts already at 3 months of age. We performed immunohistochemical analyses to compare the different sensibility of the two hippocampal regions to neurodegeneration. We performed qualitative and quantitative evaluations by fluorescence immunohistochemistry with double or triple staining, followed by confocal microscopy and digital image analysis in stratum pyramidale (SP) and stratum radiatum (SR) of CA1 and CA3, separately. We evaluated time-dependent Aβ plaques deposition, expression of inflammatory markers, as well as quantitative and morphological alterations of neurons and glia in transgenic mice at 3 (Tg 3M) and 6 (Tg 6M) months of age, compared to WT mice. In CA1 SR of Tg 6M mice, we found significantly more Medium and Large plaques than in CA3. The pattern of neurodegeneration and astrocytes activation was different in the two areas, indicating higher sensitivity of CA1. In the CA1 SP of Tg 6M mice, we found signs of reactive astrogliosis, such as increase of astrocytes density in SP, increase of GFAP expression in SR, and elongation of astrocytes branches. We found also common patterns of glia activation and neurodegenerative processes in CA1 and CA3 of Tg 6M mice: significant increase of total and reactive microglia density in SP and SR, increased expression of TNFα, of iNOS, and IL1β in astrocytes and increased density of neurons–astrocytes–microglia triads. In CA1 SP, we found decrease of volume and number of pyramidal neurons, paralleled by increase of apoptosis, and, consequently, shrinkage of CA1 SP. These data demonstrate that in TgCRND8 mice, the responses of neurons and glia to neurodegenerative patterns induced by Aβ plaques deposition is not uniform in the two hippocampal areas, and in CA1 pyramidal neurons, the higher sensitivity may be related to the different plaque distribution in this area. All these modifications may be at the basis of memory loss, the peculiar symptom of AD, which was demonstrated in this transgenic mouse model of Aβ deposition, even at early stages.

## Introduction

It is known that the pyramidal neurons of Cornu Ammonis 1 (CA1) of the hippocampus are highly sensible to insults such as ischemia, inflammation, hypoglycemia, or excitotoxicity (Whittington and Little, 1993; Prendergast et al., 2000a,b), as compared to CA3 and dentate gyrus (Bramlett et al., 1999; Kristensen et al., 2001; Lahtinen et al., 2001). While some researchers explain the higher sensitivity of CA1 neurons to the unique network properties of the hippocampal formation (Lahtinen et al., 2001) and others to the higher density of NMDA receptors in this region (Martens et al., 1998), the exact mechanisms that underlie this region-specific selective vulnerability are still not well understood. Neurons are the basic functional units of the central nervous system, but it is becoming more and more evident that glia cells are not only supportive elements, but proper functioning of the neuron-astrocyte-microglia triad is necessary for the correct organization of the brain (Barres, 2008; Allen and Barres, 2009; Cerbai et al., 2012; Lana et al., 2014, 2016, 2017). Emerging evidence indicates that inflammation and other insults can modify the neuron–astrocyte–microglia interactions (Cerbai et al., 2012; Lana et al., 2014, 2016, 2017), and this mechanism may be involved not only in brain aging but also in AD (Mercatelli et al., 2016).

Several studies have shown that microglia cells have a functional role in coordinating the responses between the immunity system and cognitive functions. During development and adulthood, microglia are critical to maintain microenvironmental homeostasis (Prinz and Priller, 2014), to perform pruning of synapses, to phagocytose apoptotic neurons and debris, and to maintain astrocyte functions (Fetler and Amigorena, 2005; Fitzner et al., 2011; Paolicelli et al., 2011; Schafer et al., 2012; Boche et al., 2013; Kettenmann et al., 2013; Parkhurst et al., 2013; Schafer and Stevens, 2013; Wake et al., 2013; Safaiyan et al., 2016). Different acute or chronic neuroinflammatory or neurodegenerative conditions can cause rapid recruitment of microglia to the site of injury, with resultant functions characteristic of macrophages, such as phagocytosis, antigen presentation, and release of immunomodulatory factors resident responses (Hanisch and Kettenmann, 2007; Prinz et al., 2011; Prinz and Priller, 2014). In the last few years, the original definition of microglia activation as a stereotypic harmful process has been disputed and revised. It is now clear that microglia respond with a variety of different reactions by integrating multifarious inputs, and microglia responses may induce neuroprotective or neurotoxic effects, depending upon the stimulus. Microglia cells are distributed throughout the brain but have differential region-specific distribution, and a wide range of morphologies in the various brain areas, such as in CA1 and CA3 of the hippocampus (Lawson et al., 1990). This heterogeneity may cause unequal sensitivity to microglia-mediated neurotoxicity and/or neuroprotection in different regions of the brain (Lawson et al., 1990). Astrocytes are cells that in physiological conditions provide trophic support to neurons. The idea that increased astrocytes is always a negative phenomenon is rapidly changing, giving way to the new concept of a more complex and more variegated role of astrocytes in different neuropathological disorders (Sofroniew, 2009; Hamby and Sofroniew, 2010; Verkhratsky et al., 2013; Burda and Sofroniew, 2014). Dysfunction in the process of astrogliosis causes loss of normal functions, increase of damaging effects, and is considered one of the causes of damaging mechanisms in the central nervous system (De Keyser et al., 2008; Farfara et al., 2008; Sofroniew, 2009). As reported by Sofroniew (2009) and Sofroniew and Vinters (2010), and further demonstrated by Cerbai et al. (2012) and Lana et al. (2014, 2016, 2017), astrogliosis is recently considered not a uniform process, nor synonymous with formation of scar tissue. In CA3 hippocampus, moderate reactive astrogliosis is found, with scarce proliferation of astrocytes that do not form scars but occupy non-overlapping domains (Bushong et al., 2002; Lana et al., 2016). Indeed, contribution of astrocytes to the pathophysiology of the brain is more complex than previously considered and glia responds with multiple cellular phenotypes that are neuroprotective at first and rarely only purely deleterious. Indeed, adaptive reactive astrogliosis may be beneficial for neurons, while suppression of astrocytes reactivity may increase neuronal vulnerability, may alter neuronal regeneration and exacerbate pathology (Sofroniew, 2009; Burda and Sofroniew, 2014; Pekny et al., 2014).

In mouse models of AD, it has been demonstrated not only increase of reactive astrocytes, especially around Aβ plaques (Bellucci et al., 2006; Giovannini et al., 2008; Rodriguez et al., 2009) but also decrease of the size of astrocytes and reduction of the number of primary branches (Olabarria et al., 2010; Yeh et al., 2011; Schitine et al., 2015). Nevertheless, astrocyte atrophy and astrogliosis in AD do not seem to occur in all brain regions to the same extent, and the possibility that astrocytes respond differently to the same stimulus in different regions of the brain, causing different outcomes on neuronal viability must be taken into consideration. Astrocytes have many fundamental roles in the brain, such as the trophic support to neurons, and significantly contribute to active mnesic-associated processes, including synaptic plasticity (Verkhratsky et al., 2011). Normally activated microglia are important as the scavenger cells of the CNS. However, proliferation and activation of microglia around Aβ plaques is a prominent feature of Alzheimer’s disease (AD) and impaired uptake and clearance of Aβ could increase the risk of developing AD (Wang et al., 2015). Indeed, when microglia fail responding to their normal regulatory feedback and/or they show an impaired ability to clear Aβ (Paresce et al., 1997; von Bernhardi, 2007), glial cells could become predominantly cytotoxic. Microglia in aged brains may have dystrophic, fragmented morphology, suggesting that AD may develop when the neuroprotective microglia function is reduced (Streit et al., 2004, 2009). There is also plenty of evidence that unbridled microglia activity can be harmful to neurons in neurodegenerative disease such as AD (Hamelin et al., 2016). Switching polarization of microglia within appropriate time windows may produce therapeutic benefits to neurodegenerative diseases.

The hippocampus, one of the brain areas involved in the mechanisms of memory (Fortin et al., 2002; Lana et al., 2013; Giovannini et al., 2015; Squire et al., 2015), is one of the regions most affected by neurodegeneration in AD. In this research we studied and compared the quantitative, temporal, and spatial modifications of the interplay between astrocytes–microglia and neurons in CA1 and CA3 hippocampus of TgCRND8 mice, a mouse model of Aβ deposition, in comparison to WT littermates. Our investigation focused on changes in areas CA1 and CA3 of the hippocampus because of their critical role in memory processing and because of the significant functional, structural, and morphological alterations in AD (Padurariu et al., 2012). A better understanding of the role of astrocytes and microglia in CA1 and CA3 regions may shed light on the mechanisms of neurodegeneration.

## Materials and Methods

### TgCRND8 mice

The TgCRND8 (Tg) mice express two mutated human APP genes implicated in AD (Swedish, KM670/672NL and Indiana, V717F) under the regulation of Syrian hamster prion promoter gene (Chishti et al., 2001). The mice were maintained on a hybrid (C57)/(C57/CH3) background by crossing transgenic heterozygous TgCRND8 males with wild-type (WT) female mice.

The main feature of this model is the very rapid development of amyloid deposition in the brain. Mice display amyloid plaques in the cortex and hippocampus already at 3 months of age (Chishti et al., 2001) since the two mutations involve both the β and γ secretase APP cleavage sites. These neuropathologic manifestations are accompanied by impaired acquisition and learning deficits (Chishti et al., 2001; Bellucci et al., 2006). The transgenic mice were generated and supplied by Dr. P. St George Hyslop (Center for Research in Neurodegenerative Diseases, Toronto, Canada), and the colony was bred in our animal house facility (Ce.S.A.L., Centro Stabulazione Animali da Laboratorio), University of Florence. All animal experiments were performed according to the Italian Law on Animal Welfare (DL 26/2014), approved by the Institutional Animal Care and Use Committee of the University of Florence and by the Italian Ministry of Health. All efforts were made to minimize animal sufferings and to use only the number of animals necessary to produce reliable scientific data. Two groups of transgenic mice were used: 3 months (*n* = 6, equally divided for sex) and 6 months old (*n* = 6, equally divided for sex). WT mice of 3 and 6 months of age (*n* = 6, equally divided for sex and age were used); since no significant differences were ever observed in any of the parameters investigated, the data from the two groups were averaged and used as controls.

At the appropriate ages (3 and 6 months), mice were deeply anesthetized with Zoletil (80 mg/kg i.p.) and were perfused transcardially with 200 ml of ice-cold paraformaldehyde solution (4% paraformaldehyde in phosphate-buffered saline, PBS, pH 7.4). After overnight post fixation and cryoprotection (18% sucrose/PBS), 40 μm-thick coronal sections were cut with a cryostat and stored at -20°C in anti-freeze solution until immunohistochemistry.

### Immunohistochemistry

Immunohistochemistry was performed with the free-floating method (Giovannini, 2002; Lana et al., 2014) on mice brain coronal sections containing the dorsal hippocampus (coordinates -1.6 to -2.0 mm from bregma, Franklin and Paxinos, 2008). The list of antibodies and their dilution is reported below. All the washings were 3 × 5 min.

#### Day 1

Free-floating sections (40 μm thick) were placed in wells of 24-well plates and were rinsed in PBS-TX and blocked for 60 min with BB (10% normal goat serum in PBS-TX and 0.05% NaN_3_). Sections were then incubated overnight at 4°C under slight agitation with a combination of two primary antibodies, both dissolved in BB.

#### Day 2

For double immunostaining, after washings, the sections were incubated for 2 h at room temperature in the dark with AlexaFluor 488-conjugated donkey anti-rabbit IgG secondary antibody diluted in BB and then for 2 h at room temperature in the dark with AlexaFluor 488-conjugated donkey anti-rabbit IgG secondary antibody plus AlexaFluor 555 goat anti-mouse both diluted 1:400 in BB.

For triple immunostaining, after washings, the sections were incubated for 2 h at room temperature in the dark with AlexaFluor 555 donkey anti-mouse IgG (1:400) secondary antibody diluted in BB and then for 2 h at room temperature in the dark with AlexaFluor 555 donkey anti-mouse IgG (1:400) plus either one of the following secondary antibodies: AlexaFluor 635 goat anti-rabbit IgG (1:400). After washings, astrocytes were immunostained using a mouse anti-GFAP antibody conjugated with the fluorochrome AlexaFluor 488, dilution 1:500, while neurons were immunostained with mouse anti-NeuN antibody conjugated with the fluorochrome AlexaFluor 488, dilution 1:500.

After extensive washings, the sections were mounted onto gelatin-coated slides using Vectashield with DAPI (Vector Laboratories).

Slices were observed under an epifluorescent microscope or a confocal laser scanning microscope (see below).

#### Antibodies

The following primary antibodies were used. For β amyloid plaques immunostaining: a rabbit anti-β amyloid 1-42 antibody, dilution 1:150 (Product Code #8243P, Cell Signaling, Danvers, MA, United States); a mouse anti-β amyloid 1-16 (6E10) antibody, dilution 1:400 (Product Code #SIG-39320, Covance, Emeryville, CA, United States). For neurons: a mouse anti-neuronal nuclei (NeuN) antibody, dilution 1:200 (Product Code #MAB377, Millipore, Billerica, MA, United States); a mouse anti-NeuN antibody conjugated with the fluorochrome AlexaFluor 488, dilution 1:500 (Product Code #MAB377X, Millipore, Billerica, MA, United States). For astrocytes: a rabbit anti-glial fibrillary acidic protein (GFAP) antibody, dilution 1:500 (Product Code #Z0334, Dako Cytomation, Glostrup, Denmark); a mouse anti-GFAP antibody conjugated with the fluorochrome AlexaFluor 488, dilution 1:500 (Product Code #MAB3402X, Millipore, Billerica, MA, United States). For total microglia: a rabbit anti-ionized calcium binding adaptor molecule 1 (IBA1) antibody, dilution 1:300 (Product Code #016-20001, WAKO, Osaka, Japan). For reactive microglia: a mouse anti-CD68 antibody, dilution 1:100 (Product code #Ab955, AbCam, Cambridge, United Kingdom). For apoptotic cells: a mouse anti-cytochrome C (Cyt C) antibody, dilution 1:200 (Product Code #556432, Becton and Dickinson, Franklin Lakes, NJ, United States). For TNF-α: a rabbit anti-TNF-α antibody, dilution 1:500 (Product Code #PA5-19810, Thermo Fisher Scientific, Waltham, MA, United States). For iNOS: a rabbit anti-iNOS antibody, dilution 1:150 (Product Code #PA3-030A, Thermo Fisher Scientific). For IL1β: a rabbit anti- IL1β (Product code #Ab9722, AbCam, Cambridge, United Kingdom).

The following fluorescent secondary antibodies were used: AlexaFluor 488 donkey anti-rabbit, dilution 1:400 (Product Code #A21206, Thermo Fisher Scientific); AlexaFluor 555 donkey anti-mouse, dilution 1:400 (Product Code #A31570, Thermo Fisher Scientific); AlexaFluor 635 goat anti-rabbit, dilution 1:400 (Product Code #A31577, Thermo Fisher Scientific). Nuclei were stained using DAPI, contained in the mounting medium for glass slides, Vectashield (Product Code #H1200, Vector Laboratories, Burlingame, CA, United States).

### Protocol Used

#### Microscopy Techniques and Quantitative Analysis

Epifluorescence and confocal microscopy acquisitions were performed in the regions of interest (ROIs, SP, and SR of CA1 and CA3 dorsal hippocampus, separately) to acquire immunofluorescence signals. The ROIs were the proximal region for CA1 as defined by Masurkar (2018) and area CA3 as defined by Lorente and de Nó (1934), Li et al. (1994), and Amaral and Lavenex (2007).

The epifluorescence microscopy images were obtained with an Olympus BX63 microscope equipped with a Metal Halide Lamp (Prior Scientific Instruments Ltd., Cambridge, United Kingdom) and a digital camera Olympus XM 10 (Olympus, Milan, Italy).

The confocal microscopy images were obtained with a LEICA TCS SP5 confocal laser scanning microscope (Leica Microsystems CMS GmbH, Mannheim, Germany. The parameters of acquisition were maintained constant: frame dimension 1240 × 1240 points, frequency of acquisition 200 Hz.

Two experimenters performed all quantitative analyses blind, and data were averaged. All evaluations of cell density were made on z projections of 10 consecutive confocal scans (total thickness 15 μm). Cells were counted, and the area of analysis was measured. Cells were expressed as density (number/mm^2^).

We performed the following quantitative analyses using ImageJ software (National Institute of Health^1^) separately in the stratum pyramidale (SP) and stratum radiatum (SR) of CA1 and CA3.

Density of cells (neurons, astrocytes, microglial cells, TNF-α, iNOS, and IL1β-positive cells) was calculated as cells/mm^2^ in SP and SR of CA1 and CA3, on confocal *z* projections of five scans (total 7.5 μm inside the section).

Aβ load was calculated as total plaque density in SP or SR of CA1 and CA3 (plaques/mm^2^). Plaques were further subdivided by size into Small (S, below 2500 μm^3^), Medium (M, between 2500 and 7000 μm^3^), and large (L, over 7000 μm^3^) and counted in SP or SR of CA1 and CA3.

For the evaluation of the volume of pyramidal neurons, we considered the neuron as a spheroid. We measured the *x* and *y* axes of five neurons chosen randomly in three different confocal planes equally spaced in the depth of the sections (total 30 cells/animal in CA1 and CA3, separately). The volumes of the cells were calculated, and data were averaged.

For the evaluation of the thickness of CA1 and CA3 SP layers, the cell layer was measured at three fixed, equidistant locations taken in three different confocal planes equally spaced in the depth of the sections (total nine measures in CA1 and CA3, separately), and data were averaged.

For the evaluation of the density of apoptotic neurons in CA1 and CA3 SP, every neuron (identified by NeuN immunostaining) with a diffuse and intense Cyt C cytoplasmatic immunostaining was considered “apoptotic” (Suen et al., 2008).

For the evaluation of the length of astrocytes branches, the length of three principal branches of five astrocytes randomly chosen was measured in three different confocal planes, equally spaced in the depth of the sections (total 30 cells/animal in CA1 and CA3, separately), and data were averaged.

For the evaluation of the expression of GFAP and TNF-α immunofluorescence, the following protocol was used: a *z* projection of 10 consecutive confocal *z* scansions was done (total thickness 15 μm). Selecting an appropriate threshold of intensity of GFAP and TNF-α immunofluorescence, z-projection was converted in a black and white image using ImageJ (care was taken to maintain a fixed threshold value among sections). Black and white pixels are points of fluorescence intensity above or under the selected threshold, respectively. The ratio between the number of black pixels and the area of analysis (mm^2^) in each section was calculated, taken as quantitative expression of GFAP and TNF-α immunofluorescence, and reported on graphs.

For the evaluation of the volume of reactive microglia, we considered the body of CD68-positive microglia as a spheroid. We measured the *x* and *y* axes of 30 reactive microglia cells/animal chosen randomly in a *z*-projection of 10 consecutive confocal *z*-scans (total thickness 15 μm). The volumes of the cells were calculated, and data were averaged. Spatial orientation of IBA1^+^ microglia toward plaques was calculated counting the microglial cells with soma contacting the surface of Large plaque plus those with their soma located within 10 μm around Large plaques, as percent of total microglia cells in the ROIs.

For the evaluation of the density of the neuron–astrocyte–microglia triads, we defined “triads” any cluster of cells in which a neuron is in direct contact with a microglial cell (undergoing phagocytosis) and one (or more than one) astrocytes take contact with the neuronal body with their branches, frequently forming a scar around it. The evaluation was made on a *z* projection of 10 consecutive confocal *z* scans (total thickness 15 μm).

### Statistical Analysis

Statistical comparisons were performed using Graph Pad Prism (Graph Pad Software Inc., La Jolla, CA, United States) by Student’s *t*-test, one-way ANOVA followed by Newman–Keuls multiple comparison test (if more than two groups were compared), two-way ANOVA and three-way ANOVA followed by Bonferroni post test, or linear regression analysis, as appropriate. Significance was set at *P* < 0.05.

## Results

### Quantitative Analysis of Aβ Plaques Deposition in CA1 and CA3 Hippocampus of TgCRND8 Mice and Evaluation of Glial Response

**FIGURE 1:**
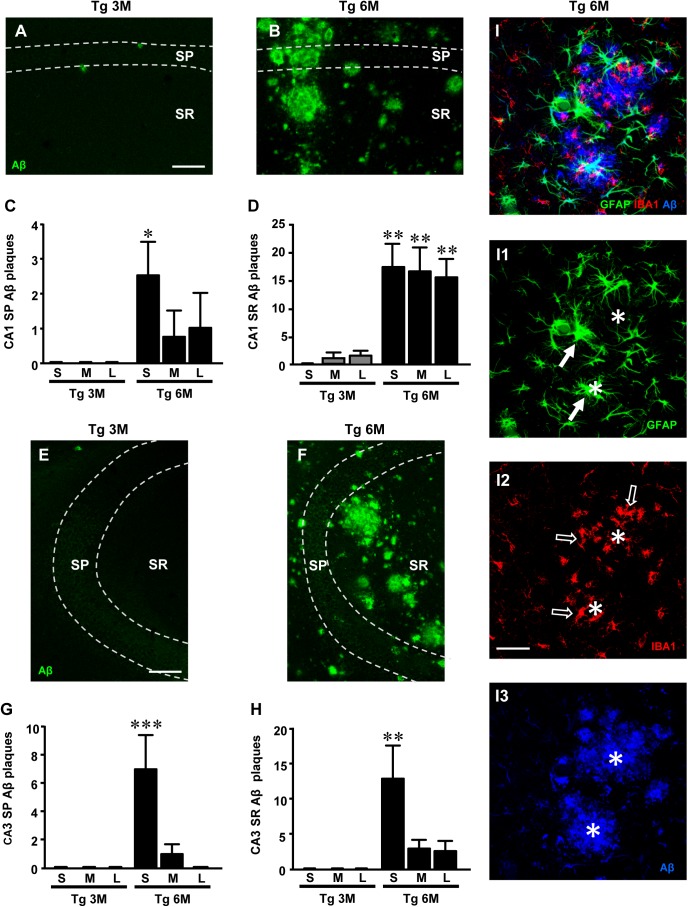
Analysis of Aβ1-42 plaques in CA1 and CA3 of Tg 3M and Tg 6M mice, and their subdivision by size into Small (S), Medium (M), and Large (L). **(A,B)** Representative confocal photomicrographs of Aβ1-42 immunostaining in plaques (green) in CA1 of a Tg 3M **(A)** and a Tg 6 M **(B)** mouse. Scale bar: 100 μm. **(C,D)** Quantitative analysis of S, M, and L plaques CA1 SP **(C)** and SR **(D)** of Tg 3M and Tg 6M. S plaques were significantly more numerous in CA1 SP of Tg 6M, while in CA1 SR of Tg 6M mice S, M, and L plaques were significantly more numerous than in Tg 3M mice. **(E,F)** Representative confocal photomicrographs of Aβ1-42 immunostaining in plaques (green) in CA3 of a Tg 3M **(E)** and a Tg 6M **(F)** mouse. Scale bar: 100μm. **(G,H)** Quantitative analysis of S, M and L plaques CA3 SP **(G)** and SR **(H)** of Tg 3M and Tg 6M. S plaques were significantly more numerous in CA3 SP and CA3 SR of Tg 6M than in Tg 3M mice. **(I–I3)** Representative confocal photomicrographs of triple immunostaining of astrocytes [**(I1)**, green], microglia [**(I2)**, red], and Aβ plaques [**(I3)**, blue] in CA1 of a Tg 6M **(B)** mouse. The merge is shown in panel **I**. Scale bar: 40 μm. Data reported in all graph bars are expressed as mean ± SEM.

We visualized the plaques by immunohistochemistry using an anti Aβ1-42 antibody (green) in hippocampal sections of TgCRND8 at 3 (Tg 3M) and 6 months of age (Tg 6M). No plaques were ever found in CA1 and CA3 hippocampus of WT mice. Images of fluorescent immunostaining were taken in CA1 (Figures 1A,B) and CA3 hippocampal regions (Figures 1E,F) with confocal microscopy and the quantitative analysis of the density of Aβ (plaques/mm^2^) was performed separately in Str. Pyramidale (SP) and Str. Radiatum (SR).

The panels in Figures 1A,B,E,F show the images of plaques (green) in CA1 SP and SR and CA3 SP and SR, respectively. Quantitative analysis showed that in CA1 SP (65.8 ± 19.2 plaques/mm^2^) and SR (247.9 ± 101 plaques/mm^2^) of Tg 6M, the density of Aβ plaques was significantly higher than in SP (1.0 ± 0.4 plaques/mm^2^) and SR (10.1 ± 7.8 plaques/mm^2^) of Tg 3M, respectively. The statistical analysis was performed by Student’s *t*-test in CA1 SP (*P* < 0.05, Tg 6M vs. Tg 3M; Tg 3M: *n* = 4; Tg 6M: *n* = 4) and in CA1 SR (*P* < 0.05, Tg 6M vs. Tg 3M; Tg 3M: *n* = 4; Tg 6M: *n* = 4).

Quantitative analysis showed that in CA3 SP (62.4 ± 28.3 plaques/mm^2^) and SR (134.5 ± 46.8 plaques/mm^2^) of Tg 6M, the density of Aβ plaques was significantly higher than in SP (0.5 ± 0.25 plaques/mm^2^) and SR (0.5 ± 0.25 plaques/mm^2^) of Tg 3M. The statistical analysis was performed by Student’s *t*-test in CA2 SP (*P* < 0.05, Tg 6M vs. Tg 3M; Tg 3M: *n* = 4; Tg 6M: *n* = 4) and in CA3 SR (Student’s *t*-test: ^∗^*P* < 0.05, Tg 6M vs. Tg 3M; Tg 3M: *n* = 4; Tg 6M: *n* = 5).

The plaques were then further characterized and quantified subdividing them by size into small (S, less than 2500 μm^3^), medium (M, between 2500 and 7000 μm^3^), and large (L, more than 7000 μm^3^) in SP or SR of CA1 and CA3, as shown in Figures 1C,D,G,H. Two-way ANOVA statistical analysis on Aβ plaques in CA1 SP of Tg 3M and Tg 6M revealed a significant main effect for experimental group [*F*(1,18) = 7.156, *P* < 0.05], no significant effect for plaque size [*F*(2,18) = 1.084, *P* > 0.05], and Interaction [*F*(2,18) = 1.084, *P* > 0.05]. Bonferroni post test showed that small plaques (S) in CA1 SP of Tg 6M were significantly more numerous than in Tg 3M (^∗^*P* < 0.05 Tg 6M S vs. Tg 3M S). Two-way ANOVA statistical analysis on Aβ plaques in CA1 SR of Tg 3M and Tg 6M revealed a significant main effect for experimental group [*F*(1,18) = 45.69, *P* < 0.001], no significant effect for plaque size [*F*(2,18) = 0.003925, *P* > 0.05], and Interaction [*F*(2,18) = 0.1647, *P* > 0.05]. Bonferroni post test showed that small (S), medium (M), and large plaques (L) in CA1 SR of Tg 6M were significantly more numerous than in Tg 3M (^∗∗^*P* < 0.01 Tg 6M S, M, L vs. Tg 3M S, M, L). Two-way ANOVA statistical analysis on Aβ plaques in CA3 SP of Tg 3M and Tg 6M revealed a significant main effect for experimental group [*F*(1,18) = 10.04, *P* > 0.01], plaque size [*F*(2,18) = 6.774, *P* > 0.01], and Interaction [*F*(2,18) = 6.774, *P* > 0.01]. Bonferroni post test showed that small plaques (S) in CA3 SP of Tg 6M were significantly more numerous than in Tg 3M (^∗∗∗^*P* < 0.01 Tg 6M S vs. Tg 3M S). Two way ANOVA statistical analysis on Aβ plaques quantification in CA3 SR of Tg 3M and Tg 6M revealed a significant main effect for experimental group [*F*(1,18) = 13.36, *P* < 0.01], plaque size [*F*(2,18) = 3.965, *P* < 0.05], and Interaction [*F*(2,18) = 3.965, *P* < 0.05]. Bonferroni post test showed that Small plaques (S) in CA3 SR of Tg 6M were significantly more numerous than in Tg 3M (^∗∗^*P* < 0.01 Tg 6M S vs. Tg 3M S).

Statistical analysis with three-way ANOVA with Plaque Size, ROI, and Age as the three variables was performed between CA1 SP and CA3 SP of Tg 3M and Tg 6M. Results showed a significant main effect for Age [*F*(1,47) = 18.01, *P* < 0.001], for Size [*F*(2,47) = 7.77, *P* < 0.01], and for Interaction Age × Size (*F*(2,47) = 7.77, *P* < 0.01). Bonferroni post test showed that Small plaques (S) in CA3 SP of Tg 6M were on average significantly more numerous than in CA1 SP of Tg 6M mice (*P* < 0.05), while Medium (M) and Large (L) plaques were not significantly different.

Statistical analysis with three way ANOVA with Plaque Size, ROI, and Age as the three variables was performed between CA1 SR and CA3 SR of Tg 3M and Tg 6M. Results showed a significant main effect for ROI [*F*(1,47) = 13.16, *P* < 0.01], Age [*F*(1,47) = 77.49, *P* < 0.001], for Interaction Age × ROI [*F*(1,47) = 9.02, *P* < 0.01], and for Interaction Age × Size [*F*(2,47) = 3.63, *P* < 0.05]. Bonferroni post test showed that Medium plaques (M) and Large plaques (L) in CA1 SR of Tg 6M were on average significantly more numerous than in CA3 SR of Tg 6M mice (*P* < 0.05), while Small plaques were not significantly different.

We performed triple immunostaining of astrocytes with GFAP (green), of microglia with IBA1 (red), and of plaques using an Aβ1-16 antibody (blue) in area CA1 of a TgCRND8 mouse at 6 months of age. The confocal image of the triple immunostaining in Figure 1I shows that Aβ plaques (Figure 1I3, asterisks) were surrounded and infiltrated by many hypertrophic astrocytes (Figure 1I1, arrows) and microglia cells (Figure 1I2, open arrows). Astrocytes and microglia located more distantly from the plaques were in a less reactive state.

We then performed a thorough investigation of the quantitative, temporal, and spatial modifications of the interplay between astrocytes–microglia and neurons in CA1 and CA3 hippocampus of TgCRND8 mice in comparison to WT littermates.

### Characterization of Astrocytes in CA1 and CA3 Hippocampus of TgCRND8 Mice

**FIGURE 2:**
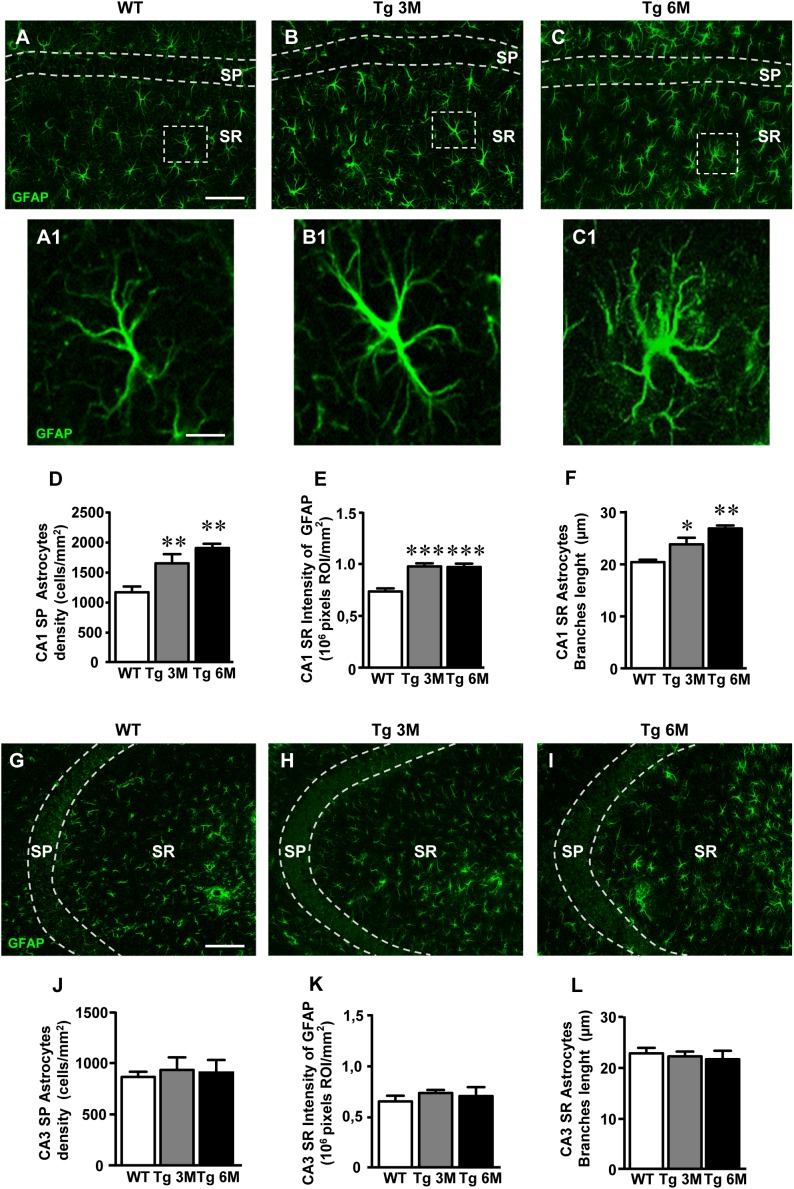
Analysis of astrocytes in CA1 and CA3 of WT, Tg 3M, and Tg 6M. **(A–C1)** Representative confocal photomicrographs of GFAP immunostaining of astrocytes (green) in CA1 of a WT **(A)**, a Tg 3M **(B)**, and a Tg 6M **(C)**. Scale bar: 100 μm. panels **(A1–C1)** shows the magnification of astrocytes framed in panels **(A–C)**. Scale bar: 15 μm. **(D)** Quantitative analysis of GFAP-positive astrocytes/mm^2^ in CA1 SP of WT, Tg 3M, and Tg 6M. Astrocytes were significantly more numerous in CA1 SP of Tg 3M and Tg 6M vs. WT mice. **(E)** Quantitative analysis of intensity of GFAP staining in CA1 SR of WT, Tg 3M, and Tg 6M. Intensity of GFAP staining was significantly increased in CA1 SR of Tg 3M and Tg 6M vs. WT mice. **(F)** Length of principal astrocyte branches in CA1 SR of WT, Tg 3M, and Tg 6M. Astrocytes branches were significantly longer in CA1 SR of Tg 3M and Tg 6M than in WT mice. **(G–I)** Representative confocal photomicrographs of GFAP immunostaining of astrocytes (green) in CA3 of a WT **(G)**, a Tg 3M **(H)**, and a Tg 6M **(I)**. Scale bar: 100 μm. **(J)** Quantitative analysis of GFAP-positive astrocytes/mm^2^ in CA3 SP of WT, Tg 3M, and Tg 6M. There are no significant differences in SP of Tg 6M and Tg 3M vs. WT mice. **(K)** Quantitative analysis of intensity of GFAP staining in CA3 SR of WT, Tg 3M, and Tg 6M. There are no significant differences in SR of Tg 3M and Tg 6M vs. WT mice. **(L)** Length of principal astrocyte branches in CA3 SR of WT, Tg 3M, and Tg 6M. There are no significant differences in SR of Tg 3M and Tg 6M vs. WT mice. Data reported in all graph bars are expressed as mean ± SEM.

For qualitative and quantitative analyses, astrocytes were visualized by immunohistochemistry with anti GFAP antibody (green) in hippocampal sections of TgCRND8 at 3 (Tg 3M) and 6 months of age (Tg 6M), and of WT control mice. Images of fluorescent immunostaining were taken in CA1 (Figures 2A–C) and CA3 hippocampal regions (Figures 2G–I) with confocal microscopy, separately. Figures 2A1–C1 show the magnification of the framed areas in the SR of WT (A), Tg 3M (B), and Tg 6M (C) mice. We performed separately in CA1 and CA3 SP and SR the qualitative and quantitative analyses to characterize the astrocytes.

The graphs in Figures 2D–F show the results of the quantitative analyses of astrocytes in CA1 SP (Figure 2D) and SR (Figures 2E,F). We found that the density of astrocytes significantly increased in SP of Tg mice both at 3 (+43%) and 6 months (+65%) of age in comparison to WT mice (Figure 2D) [one-way ANOVA: *F*(2,14) = 10.69, *P* = 0.0015; Newman–Keuls post-test: ^∗∗^*P* < 0.01 Tg 3M vs. WT, Tg 6M vs. WT; WT: *n* = 5; Tg 3M: *ns* = 6; Tg 6M: *n* = 6]. Nevertheless, in CA1 SR, we found no statistically significant difference of astrocytes density among the three experimental groups [WT: 873.0 ± 3.74, Tg 3M: 912.9 ± 41.53, Tg 6M: 860.1 ± 36.24; One-way ANOVA: *F*(2,14) = 0.615, *P* = 0.5543].

Qualitative analysis (Figures 2A1–C1) showed that in CA1 SR of Tg 3M and Tg 6M astrocytes, although not more numerous than in WT mice, had a different morphology from those present in the SR of WT mice. We thus performed further analyses on immunofluorescence intensity of GFAP and on the length of primary astrocyte branches to compare the morphology of astrocytes among the three experimental groups. We found that GFAP immunofluorescence significantly increased, in CA1 SR of Tg 3M in comparison to WT mice (+33%), reaching a plateau in CA1 SR of Tg 6M (+32%) (Figure 2E) [one-way ANOVA: *F*(2,11) = 19.26, *P* = 0.0003; Newman–Keuls post-test: ^∗∗∗^*P* < 0.001 Tg 3M vs. WT, Tg 6M vs. WT; WT: *n* = 4; Tg 3M: *n* = 6; Tg 6M: *n* = 4]. Also, primary branches of astrocytes in CA1 SR of Tg 6M were significantly longer (+24%) than in WT mice (Figure 2F) [one-way ANOVA: *F*(2,13) = 3.622, *P* = 0.0562; Newman–Keuls post-test: ^∗^*P* < 0.05 Tg 6M vs. WT; WT: *n* = 4; Tg 3M: *n* = 6; Tg 6M: *n* = 4]. Also, primary branches of astrocytes in CA1 SR of Tg 3M (+17%) and Tg 6M (+32%) were both significantly longer than in WT mice (Figure 2F) [one-way ANOVA: *F*(2,12) = 9.819, *P* = 0.003; Newman–Keuls post-test: ^∗^*P* < 0.05 Tg 3M vs. WT; ^∗∗^*P* < 0.01 Tg 6M vs. WT; WT: *n* = 5; Tg 3M: *n* = 6; Tg 6M: *n* = 4].

On the contrary, in CA3, no statistically significant differences were detected in the density of astrocytes in transgenic mice SP at both 3 months (+8% vs. WT) and 6 months (+5% vs. WT) of age (Figure 2J) [one-way ANOVA: *F*(2,14) = 0.089, *P* = 0.09150, n.s.; WT: *n* = 5; Tg 3M: *n* = 6; Tg 6M: *n* = 6]. In addition, the expression of GFAP protein in astrocytes of CA3 SR was only slightly but not significantly increased at both 3 months (+13% vs. WT) and 6 months (+8% vs. WT) of age (Figure 2K) [one-way ANOVA: *F*(2,9) = 0.570, *P* = 0.5843, n.s.; WT: *n* = 5; Tg 3M: *n* = 4; Tg 6M: *n* = 3].

In CA3 SR, we found no statistically significant difference of astrocytes density among the three experimental groups [WT: 1308.0 ± 68.30, Tg 3M: 901.8 ± 106.2, Tg 6M: 1023.0 ± 198.8; one-way ANOVA: *F*(2,15) = 1.675, *P* = 0.2205, n.s.]. The length of astrocyte branches in CA3 SR of transgenic mice was not statistically different from WT mice at both 3 months (-3%) and 6 months (-5%) of age (Figure 2L) [one-way ANOVA: *F*(2,12) = 0.2175, *P* = 0. 8076, n.s..; WT: *n* = 5; Tg 3M: *n* = 6; Tg 6M: *n* = 4].

We compared the results obtained in CA1 to those obtained in CA3 by two-way ANOVA with ROIs and experimental groups as the two variables. The statistical analysis on SP astrocytes density revealed that in WT animals there was no significant difference between CA1 and CA3 SP while a significant increase was found in CA1 SP of Tg 3M and Tg 6M. Indeed, we found a significant main effect for ROIs [(*F*(1,28) = 55.69, *P* < 0.001], experimental groups [*F*(2,28) = 6.626, *P* < 0.01], and a significant Interaction [*F*(2,28) = 5.077, *P* < 0.05]. Bonferroni post test showed that astrocytes density in CA1 SP of Tg 3M (*P* < 0.001 vs. CA3 SP) and Tg 6M (*P* < 0.001 vs. CA3 SP) was significantly higher than in CA3 SP.

The statistical analysis on GFAP immunofluorescence revealed that in WT animals, there was no significant difference between CA1 and CA3 SR while a significant increase was found in CA1 SR of Tg 3M and Tg 6M. Indeed, we found a significant main effect for ROIs [*F*(1,20) = 29.66, *P* < 0.001], experimental groups [*F*(2,20) = 7.654, *P* < 0.01], while the Interaction was not significant [*F*(2,20) = 2.437, n.s.]. Bonferroni post test showed that GFAP immunofluorescence in CA1 SR of Tg 3M (*P* < 0.01 vs. CA3 SR) and Tg 6M (*P* < 0.01 vs. CA3 SR) was significantly higher than in CA3 SR.

The statistical analysis on the length of primary astrocytes branches revealed that in WT and Tg 3M animals there was no significant difference between CA1 and CA3 SR while a significant increase was found in CA1 SR of Tg 6M. Indeed, we found a significant main effect for Interaction [*F*(2,24) = 6.044, *P* < 0.01]. Bonferroni post test showed that the length of primary astrocytes branches in CA1 SR of Tg 6M was significantly higher than in CA3 SR (*P* < 0.05).

### Quantitative Analysis of Total and Reactive Microglia in CA1 and CA3 Hippocampus of TgCRND8 Mice

**FIGURE 3:**
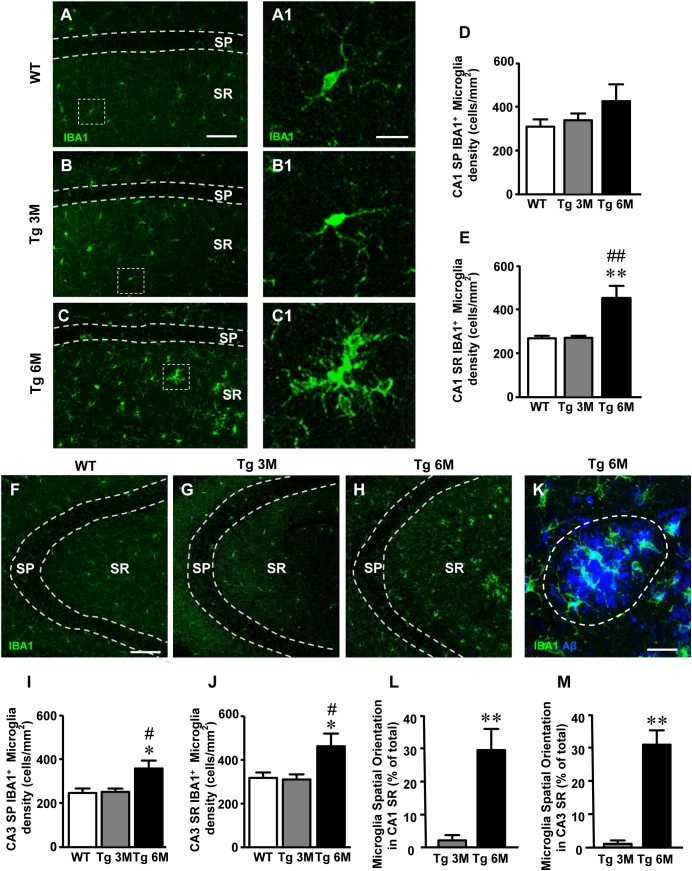
Analysis of total microglia in CA1 and CA3 of WT, Tg 3M, and Tg 6M. **(A–C)** Representative confocal photomicrographs of IBA1 immunostaining of microglia (green) in CA1 of a WT **(A)**, a Tg 3M **(B)**, and a Tg 6M **(C)**. Scale bar: 100 μm. Panels **(A1–C1)** shows the magnification of microglial cells framed in panels **(A–C)**. Scale bar: 20 μm. **(D,E)** Quantitative analysis of microglia/mm^2^ in CA1 SP **(D)** and SR **(E)** of WT, Tg 3M, and Tg 6M. Microglia cells are significantly more numerous in CA1 SR of Tg 6M mice vs. WT and Tg 3M. **(F–H)** Representative confocal photomicrographs of IBA1 immunostaining of microglia (green) in CA3 of a WT **(F)**, a Tg 3M **(G)**, and a Tg 6M **(H)**. Scale bar: 100 μm. **(I,J)** Quantitative analysis of microglia/mm^2^ in CA3 SP **(I)** and SR **(J)** of WT, Tg 3M, and Tg 6M. Microglia cells are significantly more numerous in SP and SR of Tg 6M mice vs. WT and Tg 3M. **(K)** Representative confocal image of microglia cells (green) spatially oriented toward a Large Aβ1-42 plaque (blue). The dotted line represents the area used for the quantitative analysis of spatially oriented microglia. Scale bar: 20 μm. **(L,M)** Quantitative analysis of spatially oriented microglia in CA1 SR **(L)** and CA3 SR **(M)**, represented as microglia located on or within 10 μm around Large plaques (area enclosed in the dotted line in panel **K** expressed as percent of total microglia. In both CA1 and CA3 SR of Tg 6M mice, the percent of microglia spatially oriented toward Large plaques was significantly higher than in Tg 3M. Data reported in all graph bars are expressed as mean ± SEM.

To perform the quantitative analysis of total microglia on hippocampal sections of TgCRND8 (Tg 3M, Tg 6M) and control mice (WT), we immunolabelled microglia with anti IBA1 antibody. Images of fluorescent immunostaining were taken in CA1 (Figures 3A–C) and CA3 hippocampal regions (Figures 3F–H) with confocal microscopy. Figures 3A1–C1 show the magnification of the framed areas in the SR of WT (A), Tg 3M (B), and Tg 6M (C) mice. The quantitative analyses of total microglia were performed in CA1 and CA3 SP and SR, separately.

The graphs in Figures 3D,E show the results of the quantitative analyses of the density of IBA1^+^ microglia in CA1 SP and CA1 SR. In CA1 SP of Tg 6M, we found a slight (+35%), although not statistically significant, increase of the density of IBA1^+^ microglia in comparison to WT mice (Figure 3D) [one-way ANOVA: *F*(2,14) = 1.252, *P* = 0.3161, n. s.; WT: *n* = 6; Tg 3M: *n* = 5; Tg 6M: *n* = 6]. However, the density of IBA1^+^ microglia in CA1 SR of Tg 6M significantly increased both in comparison to WT and to Tg 3M (+72% vs. WT; +43% vs. Tg 3M; Figure 3E) [one-way ANOVA: *F*(2,13) = 11.23, *P* = 0.0015; Newman–Keuls post-test: ^∗∗^*P* < 0.01 Tg 6M vs. WT, ^##^*P* < 0.01 Tg 6M vs. Tg 3M; WT: *n* = 6; Tg 3M: *n* = 5; Tg 6M: *n* = 5].

In CA3 SP of Tg 6M mice, we found a statistically significant increase of the density of IBA1^+^ microglia in comparison both to WT and Tg 3M mice (+46% vs. WT; +43% vs. Tg 3M; Figure 3I) [one-way ANOVA: *F*(2,13) = 6.217, *P* = 0.0127; Newman–Keuls post-test: ^∗^*P* < 0.05 Tg 6M vs. WT, ^#^*P* < 0.05 Tg 6M vs. Tg 3M; WT: *n* = 6; Tg 3M: *n* = 5; Tg 6M: *n* = 5]. IBA1^+^ microglia density increased also in CA3 SR of Tg 6M and the effect was statistically significant both from WT and Tg 3M mice (+48% vs. WT; +51% vs. Tg 3M; Figure 3J) [one-way ANOVA: *F*(2,13) = 5.570, *P* = 0.0179; Newman–Keuls post-test: ^∗^*P* < 0.05 Tg 6M vs. WT, ^#^*P* < 0.05 Tg 6M vs. Tg 3M; WT: *n* = 6; Tg 3M: *n* = 5; Tg 6M: *n* = 5].

Two-way ANOVA analysis demonstrated that IBA1^+^ microglia density was not significantly different in CA1 SP and SR in comparison to CA3 SP and SR of all groups examined.

We studied the spatial orientation of IBA1^+^ microglia toward Large plaques calculating the percent microglia cells located on or within 10 μm around Large plaques in CA1 and CA3 SR (see dotted line in Figure 3K which shows a Large plaque in blue and microglia cells in green). Quantitative analysis showed that in CA1 and CA3 SR of TG 6M mice a highly significant percent of microglia was oriented toward Large plaques in comparison to TG 3M (^∗∗^*P* < 0.01, Tg 6M vs. Tg 3M, Student’s *t*-test, Figures 3L,M). Two-way ANOVA analysis demonstrated that the orientation of IBA1^+^ microglia toward Large plaques was not significantly different in CA1 SR in comparison to CA3 SR of all groups examined.

**FIGURE 4:**
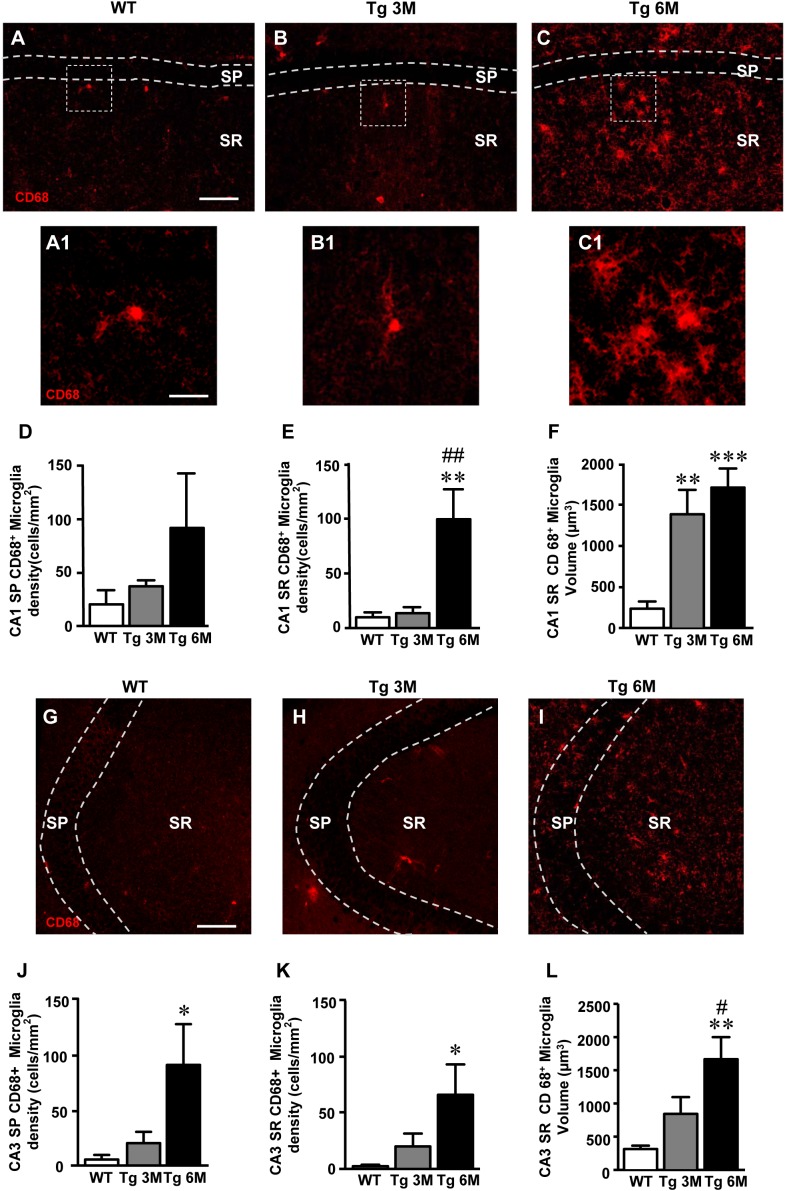
Analysis of reactive microglia in CA1 and CA3 of WT, Tg 3M, and Tg 6M. **(A–C)** Representative confocal photomicrographs of CD68 immunostaining of reactive microglia (red) in CA1 of a WT **(A)**, a Tg 3M **(B)**, and a Tg 6M **(C)**. Scale bar: 100 μm. Panels **(A1–C1)** shows the magnification of reactive microglial cells framed in panels **(A–C)**. Scale bar: 20 μm. **(D,E)** Quantitative analysis of reactive microglia/mm^2^ in CA1 SP **(D)** and CA1 SR **(E)** of WT, Tg 3M, and Tg 6M. Microglia cells are significantly more numerous in CA1 SR of Tg 6M mice vs. WT and Tg 3M. **(F)** Measure of reactive microglia cells volume in CA1 SR **(F)** of WT, Tg 3M, and Tg 6M. Reactive microglia cells volume was significantly increase in Tg 6M and Tg 3M vs. WT. **(G–I)** Representative confocal photomicrographs of CD68 immunostaining of reactive microglia (red) in CA3 of a WT **(G)**, a Tg 3M **(H)**, and a Tg 6M **(I)**. Scale bar: 100 μm. **(J,K)** Quantitative analysis of microglia/mm^2^ in CA3 SP **(J)** and SR **(K)** of WT, Tg 3M and Tg 6M. Microglia cells are significantly more numerous in SP and SR of Tg 6M mice vs. WT. **(L)** Measure of reactive microglia cells volume in CA3 SR **(L)** of WT, Tg 3M, and Tg 6M. Reactive microglia cells volume was significantly increase in Tg 6M vs. WT. Data reported in all graph bars are expressed as mean ± SEM.

To visualize reactive microglia, we performed the immunolabeling with anti CD68 antibody, a marker of reactive microglia cells, on hippocampal sections of TgCRND8 (Tg 3M, Tg 6M) and control mice (WT). Images of fluorescent immunostaining were taken in CA1 (Figures 4A–C) and CA3 hippocampal regions (Figures 4G–I) with confocal microscopy and the quantitative analysis of the density of reactive microglia was performed in CA1 and CA3 SP and SR, separately. Figures 6A1–C1 show the magnification of the framed areas in the SR of WT (A), Tg 3M (B), and Tg 6M (C) mice.

The graphs in Figures 4D,E show the results of the quantitative analyses of reactive microglia in CA1 SP and CA1 SR, respectively. In CA1 SP of Tg 3M and Tg 6M, the density of reactive microglia increased (+82 and +347% vs. WT, respectively) although not significantly, in comparison to WT mice (Figure 4D) [one-way ANOVA: *F*(2,14) = 1.395, *P* = 0.2802, n. s.; WT: *n* = 6; Tg 3M: *n* = 5; Tg 6M: *n* = 6]. On the contrary, in CA1 SR of Tg 6M, we found a highly significant increase of the density of microglia in comparison both to WT and Tg 3M mice (+902% vs. WT; +636 vs. Tg 3M; Figure 4E) [one-way ANOVA: *F*(2,13) = 10.77, *P* = 0.0017; Newman–Keuls post-test: ^∗∗^*P* < 0.01 Tg 6M vs. WT, ^##^*P* < 0.01 Tg 6M vs. Tg 3M; WT: *n* = 6; Tg 3M: *n* = 5; Tg 6M: *n* = 5]. We evaluated the volume of reactive microglia in CA1 SR, and we found a statistically significant increase of the average volume of CD68^+^ microglia in CA1 SR both at 3 (+487% vs. WT) and 6 months (+626% vs. WT) of age, in comparison to WT mice (Figure 4F) [one-way ANOVA: *F*(2,14) = 13.84, *P* = 0.0005; Newman–Keuls post-test: ^∗∗^*P* < 0.01 Tg 3M vs. WT, ^∗∗∗^*P* < 0.001 Tg 6M vs. WT; WT: *n* = 6; Tg 3M: *n* = 5; Tg 6M: *n* = 6].

Results obtained in CA3 (Figures 4J–L) are in accordance with those in CA1. In CA3 SP, we found a statistically significant increase of the density of reactive microglia in Tg 6M in comparison to WT and Tg 3M mice (+1448% vs. WT; +339% vs. Tg 3M; Figure 4J) [one-way ANOVA: *F*(2,14) = 4.002, *P* = 0.0422; Newman–Keuls post-test: ^∗^*P* < 0.05 Tg 6M vs. WT; WT: *n* = 6; Tg 3M: *n* = 5; Tg 6M: *n* = 6]. In addition, in CA3 SR of Tg 6M mice reactive microglia significantly increased in comparison to WT (+3395%) (Figure 4K) [one-way ANOVA: *F*(2,13) = 4.244, *P* = 0.0381; Newman–Keuls post-test: ^∗^*P* < 0.05 Tg 6M vs. WT; WT: *n* = 6; Tg 3M: *n* = 5; Tg 6M: *n* = 5]. The evaluation of the volume of reactive microglia in CA3 SR showed that there was a statistically significant increase of the average volume of CD68^+^ microglia in CA3 SR at 6 months (434% vs. WT) of age, in comparison to WT mice (Figure 4L) [one-way ANOVA: *F*(2,14) = 8.350, *P* = 0.0041; Newman–Keuls post-test: ^∗∗^*P* < 0.01 Tg 6M vs. WT, ^#^*P* < 0.05 Tg 6M vs. Tg 3M; WT: *n* = 6; Tg 3M: *n* = 5; Tg 6M: *n* = 6].

Two-way ANOVA analysis demonstrated that the CD68^+^ microglia density was not significantly different in CA1 SP and SR in comparison to CA3 SP and SR of all groups examined.

### Analysis of Inflammatory Mediators in CA1 and CA3 Hippocampus of TgCRND8 Mice

**FIGURE 5:**
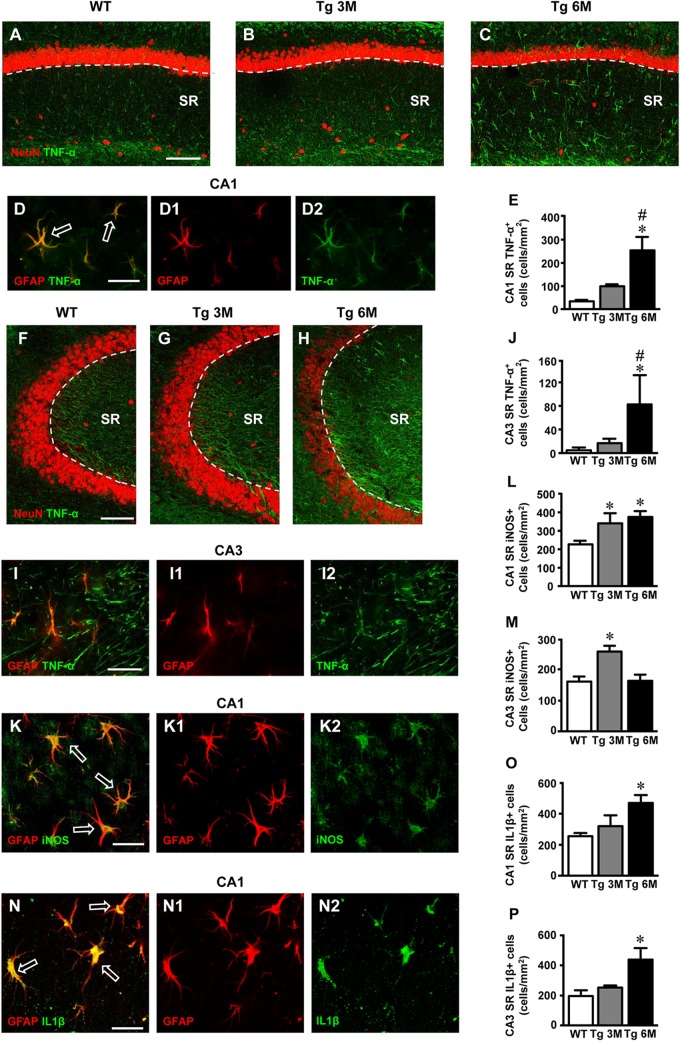
Analysis of TNF-α cellular expression in CA1 and CA3 of WT, Tg 3M, and Tg6M. **(A–C)** Representative confocal photomicrographs of TNF-α immunostaining (green) and NeuN immunostaining of neurons (red) in CA1 of a WT **(A)**, a Tg 3M **(B)**, and a Tg 6M **(C)**. Scale bar: 100 μm. **(D–D2)** Double staining immunohistochemistry with anti TNF-α (green) and anti GFAP (red) antibodies. In CA1 SR we obtained a near complete colocalization of TNF-α on astrocytes (GFAP-positive cells). Scale bar: 25 μm. **(E)** Quantitative analysis of TNF-α^+^ cells/mm^2^ in CA1 SR. TNF-α^+^ cells were significantly more numerous in SR of Tg 6M vs. WT mice. **(F–H)** Representative confocal photomicrographs of TNF-α immunostaining (green) and NeuN immunostaining of neurons (red) in CA3 of a WT **(H)**, a Tg 3M **(I)**, and a Tg 6M **(J)**. Scale bar: 100 μm. **(I–I2)** Double staining immunohistochemistry with anti TNF-α (green) and anti GFAP (red) antibodies in CA3 SR of Tg 6M mice. We found partial colocalization of TNF-α in astrocytes. Scale bar: 25 μm. **(J)** Quantitative analysis of TNF-α^+^ cells/mm^2^ in CA3 SR. TNF-α^+^ cells were significantly more numerous in SR of Tg 6M vs. WT mice. **(K–K2)** Double staining immunohistochemistry with anti iNOS (green) and anti GFAP (red) antibodies in CA1 SR of Tg 6M mice. We found colocalization of iNOS in astrocytes. Scale bar: 25 μm. **(L)** Quantitative analysis of iNOS^+^ cells/mm^2^ in CA1 SR. iNOS^+^ cells were significantly more numerous in SR of Tg 3M and Tg 6M vs. WT mice. **(M)** Quantitative analysis of iNOS^+^ cells/mm^2^ in CA3 SR. iNOS^+^ cells were significantly more numerous in SR of Tg 3M vs. WT mice. **(N–N2)** Double staining immunohistochemistry with anti IL1β (green) and anti GFAP (red) antibodies in CA1 SR of Tg 6M mice. We found colocalization of IL1β in astrocytes. Scale bar: 25 μm. **(O)** Quantitative analysis of IL1β^+^ cells/mm^2^ in CA1 SR. IL1β^+^ cells were significantly more numerous in SR of Tg 6M vs. WT mice. **(P)** Quantitative analysis of IL1β^+^ cells/mm^2^ in CA3 SR. IL1β^+^ cells were significantly more numerous in SR of Tg 6M vs. WT mice.

To verify whether different expression of inflammatory mediators might be present in CA1 and CA3, we first performed immunofluorescence staining with anti TNF-α antibody on hippocampal sections of TgCRND8 (Tg 3M, Tg 6M) and control mice (WT). Neurons were counterstained with anti-NeuN antibody. Images of fluorescent immunostaining were taken in CA1 (Figures 5A–C) and CA3 hippocampal regions (Figures 5F–H) with confocal microscopy and the quantitative analysis of the density of TNF-α-positive cells was performed in CA1 and CA3 SR, separately.

In CA1 of transgenic mice, TNF-α immunostaining increased and was localized mainly in astrocyte-like cells evenly scattered throughout the SR. To identify unequivocally the type of TNF-α-positive cells, we performed a double staining immunohistochemistry with anti TNF-α and anti GFAP antibodies. In CA1 SR of transgenic mice, we found a near complete colocalization of TNF-α in GFAP-positive astrocytes. Figures 5D–D2 show a group of TNF-α-positive astrocytes in the CA1 SR of a Tg 6M mouse. It is evident that all GFAP-positive astrocytes (D1, red) express TNF-α (D2, green), as evidenced by the yellow-orange color in Figure 5D. The graph in Figure 5E shows the results of the quantitative analysis of TNF-α-positive cells in CA1 SR. We found a significant increase of density of TNF-α-positive cells in CA1 SR in Tg 6M (+410% vs. WT) in comparison to WT [one-way ANOVA: *F*(2,13) = 8.712, *P* = 0.0040; Newman–Keuls post-test: ^∗∗^*P* < 0.01 Tg 6M vs. WT, ^#^*P* < 0.05 Tg 6M vs. Tg 3M; WT: *n* = 6; Tg 3M: *n* = 6; Tg 6M: *n* = 5]. In Tg 3M, we found a slight, not significant increase of TNF-α-positive cells in comparison to WT (+187% vs. WT).

In CA3 SR of transgenic mice, TNF-α immunostaining increased. The double staining immunohistochemistry with anti TNF-α (Figure 5I2, green), and anti GFAP antibodies (Figure 5I1, red) revealed that in CA3 SR of transgenic mice all GFAP-positive astrocytes expressed TNF-α as evidenced by the yellow-orange color in Figure 5I. TNF-α-positive astrocytes were quantified in CA3. The results showed that the density of TNF-α-positive astrocytes significantly increased in CA3 SR of Tg 6M in comparison to WT and to Tg 3M (+1646% vs. WT; +393% vs. Tg 3M) (Figure 5J) [one-way ANOVA: *F*(2,12) = 4.454, *P* = 0.0358; Newman–Keuls post-test: ^∗^*P* < 0.05 Tg 6M vs. WT, ^#^*P* < 0.05 Tg 6M vs. Tg 3M; WT: *n* = 6; Tg 3M: *n* = 6; Tg 6M: *n* = 3]. In CA3 SR, other structures, mainly located toward the DG were also TNF-α positive (Figure 5I2).

We compared the results obtained in CA1 to those obtained in CA3 by two-way ANOVA with ROIs and experimental groups as the two variables.

The statistical analysis on the density of TNF-α-positive cells revealed that in WT animals there was no significant difference between CA1 and CA3 SR while a significant increase was found in CA1 SR of Tg 3M and Tg 6M. Indeed, we found a significant main effect for ROIs [*F*(1,25) = 16.04, *P* < 0.001], experimental groups [*F*(2,25) = 13.94, *P* < 0.001], but no significant Interaction [*F*(2,25) = 1.326, n.s.]. Bonferroni post test showed that astrocytes density in CA1 SR of Tg 3M (*P* < 0.05 vs. CA3 SR) and Tg 6M (*P* < 0.05 vs. CA3 SP) was significantly higher than in CA3 SR.

Immunofluorescence staining with anti iNOS antibody (Figures 5K–K2, green) was performed on hippocampal sections of TgCRND8 (Tg 3M, Tg 6M) and control mice (WT). Astrocytes were counterstained with anti-GFAP antibody (Figures 5K,K1, red) and neurons with anti NeuN antibody (not shown). Images of fluorescent immunostaining were taken in CA1 (Figures 5K–K2) and CA3(not shown) hippocampal regions with confocal microscopy and the quantitative analysis of the density of iNOS-positive cells was performed in CA1 and CA3 SR, separately. Double labeling confocal microscopy with anti-iNOS (Figure 5K2) and anti-GFAP (Figure 5K1) demonstrated that in CA1 SR of Tg 6M iNOS was expressed GFAP-positive astrocytes, as evidenced by the yellow-orange color in Figure 5K (arrows). Double labeling confocal microscopy with anti-iNOS and anti-GFAP demonstrated that in CA3 SR of Tg 6M iNOS was expressed in GFAP-positive astrocytes (not shown).

We found a significant increase of iNOS expression in cells in CA1 SR of Tg 3M (+51%) and of Tg 6M (+66%) in comparison to WT. The statistical analysis was performed on the density of iNOS-positive cells in CA1 SR [one-way ANOVA: *F*(2,13) = 5.12, *P* = 0.0268; Newman–Keuls post-test: ^∗^*P* < 0.05 Tg 3M and, ^∗^*P* < 0.05 Tg 6M vs. WT; WT: *n* = 5; Tg 3M: *n* = 4; Tg 6M: *n* = 5; Figure 5L].

We found a significant increase of iNOS expression in cells of CA3 SR of Tg 3M (+58%), while in Tg 6M iNOS expression in cell was not different from control values (+1%) in comparison to WT mice. The statistical analysis was performed on the density of iNOS-positive cells in CA3 SR [one-way ANOVA: *F*(2,11) = 8.762, *P* = 0.0077; Newman–Keuls post-test: ^∗^*P* < 0.05 Tg 3M vs. Tg 6M and WT; WT: *n* = 4; Tg 3M: *n* = 4; Tg 6M: *n* = 4; Figure 5M].

Both in CA1 SR and CA3 SR iNOS was also expressed in neurons, but the effect was not different from WT mice (data not shown).

We compared the results obtained in CA1 to those obtained in CA3 by two-way ANOVA with ROIs and experimental groups as the two variables.

The statistical analysis on the density of iNOS-positive cells revealed that in WT and Tg 3M animals there was no significant difference between CA1 and CA3 SR while a significant increase was found in CA1 SR of Tg 6M. We found a significant main effect for ROIs (*F*(1,20) = 25.23, *P* < 0.001), experimental groups (*F*(2,20) = 6.513, *P* < 0.01), and for Interaction (*F*(2,20) = 3.540, *P* < 0.05). Bonferroni post test showed that the density of iNOS-positive in CA1 SR of Tg 6M was significantly higher than in CA3 SR (*P* < 0.001).

Immunofluorescence staining with anti IL1β antibody (Figures 5N,N2, green) was performed on hippocampal sections of TgCRND8 (Tg 3M, Tg 6M) and control mice (WT). Astrocytes were counterstained with anti-GFAP antibody (Figures 5N,N1, red) and microglia with anti-CD68 antibody (not shown). Images of fluorescent immunostaining were taken in CA1 (Figures 5N–N2) and CA3 hippocampal regions (not shown) with confocal microscopy and the quantitative analysis of the density of IL1β-positive cells was performed in CA1 and CA3 SR, separately. Double labeling confocal microscopy with anti-IL1β (Figure 5N2) and anti-GFAP (Figure 5N1) demonstrated that in CA1 SR of Tg 6M, IL1β was expressed in GFAP-positive astrocytes, as evidenced by the yellow-orange color in Figure 5N (arrows). Double labeling confocal microscopy with anti-IL1β and anti-GFAP demonstrated that in CA3 SR of Tg 6M, IL1β was expressed in GFAP-positive astrocytes (not shown).

We found a slight increase of IL1β expression in cells in CA1 SR of Tg 3M (+25%, not significant) and a significant increase in CA1 SR of Tg 6M (+83%) in comparison to WT mice. The statistical analysis was performed on the density of IL1β-positive cells in CA1 SR [one-way ANOVA: *F*(2,11) = 5.733, *P* = 0.0248; Newman–Keuls post-test: ^∗^*P* < 0.05 Tg 6M vs. WT; WT: *n* = 4; Tg 3M: *n* = 3; Tg 6M: *n* = 5; Figure 5O].

We found a slight significant increase of IL1β expression in cells of CA3 SR of Tg 3M (+28%, not significant), while in Tg 6M, IL1β expression in cells was significantly higher than in WT (+122%). The statistical analysis was performed on the density of IL1β-positive cells in CA3 SR [one-way ANOVA: *F*(2,11) = 4.730, *P* = 0.0394; Newman–Keuls post-test: ^∗^*P* < 0.05 Tg 6M vs. WT; WT: *n* = 4; Tg 3M: *n* = 3; Tg 6M: *n* = 5; Figure 5P].

Two-way ANOVA analysis demonstrated that IL1β expression in cells was not significantly different in CA1 SR in comparison to CA3 SR of all groups examined.

Both in CA1 SR and CA3 SR, IL1β was also expressed in microglia, but the effect was not different from WT mice (data not shown).

### Characterization of Neurons in CA1 and CA3 Pyramidal Layers in TgCRND8 Mice

**FIGURE 6:**
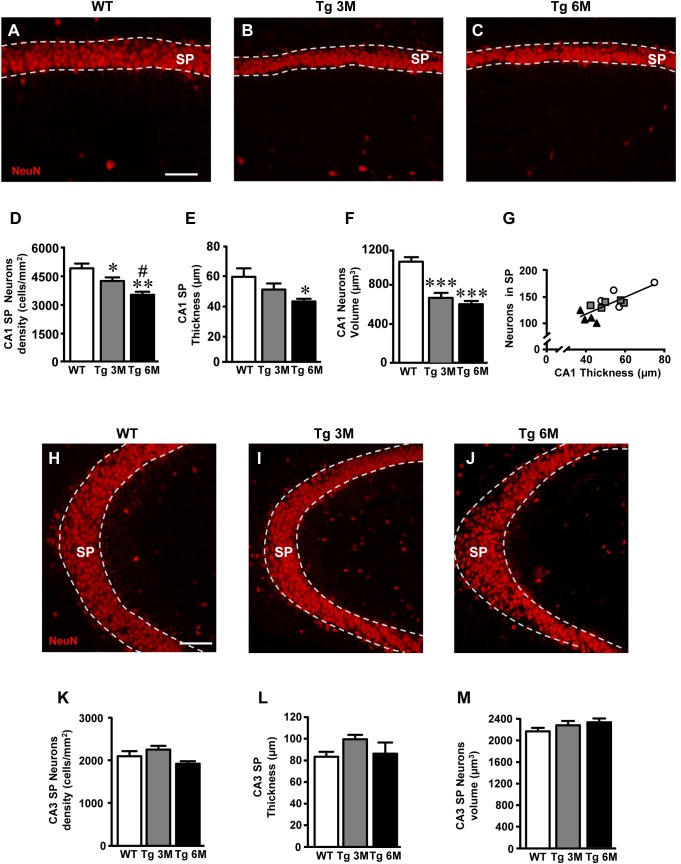
Analysis of neurons in CA1 and CA3 of WT, Tg 3M, and Tg 6M. **(A–C)** Representative confocal photomicrographs of NeuN immunostaining of neurons (red) in CA1 of a WT **(A)**, a Tg 3M **(B)**, and a Tg 6M **(C)**. Scale bar: 60 μm. **(D)** Quantitative analysis of neurons/mm^2^ in CA1 SP **(D)** of WT, Tg 3M, and Tg 6M. Neurons were significantly less numerous in SP of Tg 6M and Tg 3M vs. WT mice. **(E)** Measure of thickness of CA1 SP **(E)** of WT, Tg 3M, and Tg 6M. Thickness of CA1 SP was significantly reduced in Tg 6M vs. WT. **(F)** Measure of neurons volume in CA1 SP **(F)** of WT, Tg 3M, and Tg 6M. Neurons volume was significantly reduced in Tg 6M and Tg 3M vs. WT. **(G)** Correlation analysis between number of CA1 SP neurons and CA1 SP thickness. There is a highly significative correlation. **(H–J)** Representative confocal photomicrographs of NeuN immunostaining of neurons (red) in CA3 of a WT **(H)**, a Tg 3M **(I)**, and a Tg 6M **(J)**. Scale bar: 60 μm. **(K)** Quantitative analysis of neurons/mm^2^ in CA3 SP **(K)** of WT, Tg 3M, and Tg 6M. There are no significant differences in SP of Tg 6M and Tg 3M vs. WT mice. **(L)** Measure of thickness of CA3 SP **(L)** of WT, Tg 3M, and Tg 6M. There are no significant differences in thickness of CA3 SP of Tg 6M and Tg 3M vs. WT. **(M)** Measure of neurons volume in CA3 SP **(M)** of WT, Tg 3M, and Tg 6M. There are no significant differences in neurons volume in CA3 SP of Tg 6M and Tg 3M vs. WT. Data reported in all graph bars are expressed as mean ± SEM.

We evaluated the time-course of the extent of damage of CA1 and CA3 pyramidal neurons in Tg mice using the immunohistochemical staining of neurons with anti NeuN antibody (red) on hippocampal sections of TgCRND8 mice at 3 (Tg 3M) and 6 months of age (Tg 6M) and of WT control mice. Images of fluorescent immunostaining were taken in CA1 (Figures 6A–C) and CA3 hippocampal regions (Figures 6H–J) with confocal microscopy and the quantitative analyses of the density (Figure 6D) and the volume of pyramidal neurons (Figure 6F), and of thickness of the pyramidal layer (Figure 6E) were performed.

The graphs in Figures 6D–F show the results of the quantitative analyses in CA1. The density of neurons was significantly lower in CA1 SP of Tg mice at 3 (-15% vs. WT) and 6 months (-28% vs. WT) of age than in CA1 of WT mice (Figure 6D) [one-way ANOVA: *F*(2,12) = 9.552, *P* = 0.0033; Newman–Keuls post-test: ^∗∗^*P* < 0.01 Tg 6M vs. WT, ^∗^*P* < 0.05 Tg 3M vs. WT, ^#^*P* < 0.05 Tg 6M vs. Tg 3M; WT: *n* = 5; Tg 3M: *n* = 6; Tg 6M: *n* = 4]. Consistently, we found a reduction of CA1 SP thickness in 3 (-12%) and 6 months (-40%) old Tg mice in comparison to WT mice (Figure 6E). The effect was statistically significant at 6 months of age only [one-way ANOVA: *F*(2,10) = 4.658, *P* = 0.0372; Newman–Keuls post-test: ^∗^*P* < 0.05 Tg 6M vs. WT; WT: *n* = 4; Tg 3M: *n* = 5; Tg 6M: *n* = 4]. Also, we found a statistically significant reduction of the average volume of CA1 pyramidal neurons both at 3 (-36% vs. WT) and 6 months (-42% vs. WT) of age in comparison to WT mice (Figure 6F) [one-way ANOVA: *F*(2,12) = 23.50, *P* < 0.0001; Newman–Keuls post-test: ^∗∗∗^*P* < 0.001 Tg 3M vs. WT, Tg 6M vs. WT; WT: *n* = 5; Tg 3M: *n* = 6; Tg 6M: *n* = 4]. Correlation analysis between the number of CA1 Pyramidal neurons and the thickness of CA1 Pyramidal layer is shown in the graph in Figure 6G. We found a highly significant correlation between the two parameters (^∗∗^*P* = 0.01, *R*^2^ = 0.5763; WT: *n* = 5; Tg 3M: *n* = 6; Tg 6M: *n* = 4).

We analyzed the density of CA3 pyramidal neurons, their volume and the thickness of CA3 SP. Surprisingly, the density of CA3 pyramidal neurons in SP of Tg 3M and Tg 6M (-8% vs. WT, Figure 6K) [one-way ANOVA: *F*(2,12) = 2.933, *P* = 0.0918, n.s.; WT: *n* = 5; Tg 3M: *n* = 6; Tg 6M: *n* = 4] and the thickness of the pyramidal layer (+19% vs. WT, Figure 6L) [one-way ANOVA: *F*(2,12) = 1.897, *P* = 0.1923, n.s.; WT: *n* = 5; Tg 3M: *n* = 6; Tg 6M: *n* = 4] were not significantly different from WT mice. Also, the average volume of CA3 pyramidal neurons of transgenic mice at 3 and 6 months of age was not significantly different from WT mice (Figure 6M) [one-way ANOVA: *F*(2,12) = 1.268, *P* = 0.3165, n.s.; WT: *n* = 5; Tg 3M: *n* = 6; Tg 6M: *n* = 4], as shown by the statistical analysis, as shown by the statistical analysis.

### Quantitative Analysis of Apoptotic Neurons in CA1 and CA3 Hippocampus of TgCRND8 Mice

**FIGURE 7:**
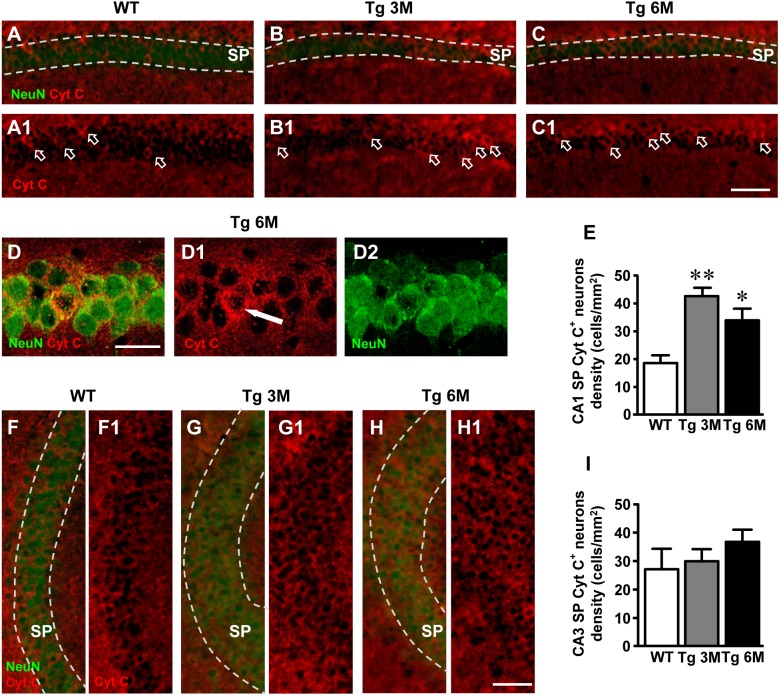
Analysis of apoptotic neurons in CA1 and CA3 of WT, Tg 3M, and Tg 6M. **(A–C1)** Representative epifluorescence photomicrographs of Cyt C immunostaining of apoptotic neurons (red) and NeuN immunostaining of neurons (green) in CA1 SP of WT **(A,A1)**, a Tg 3M **(B,B1)**, and a Tg 6M **(C,C1)**. The arrows in **(A1–C1)** point to apoptotic neurons in CA1 SP. Scale bar: 100 μm. **(D–D2)** Confocal magnification of apoptotic neurons in CA1 SP of a Tg 6M. Scale bar: 30 μm. **(E)** Quantitative analysis of apoptotic neurons/mm^2^ in CA1 SP of WT, Tg 3M, and Tg 6M. Apoptotic pyramidal neurons were significantly more numerous in SP of Tg 3M and Tg 6M vs. WT mice. **(F–H1)** Representative epifluorescence photomicrographs of Cyt C immunostaining of apoptotic neurons (red) and NeuN immunostaining of neurons (green) in CA3 SP of WT **(F,F1)**, a Tg 3M **(G,G1)** and a Tg 6M **(H,H1)**. The arrows in **(F1–H1)** point to apoptotic neurons in CA3 SP. Scale bar: 100 μm. **(I)** Quantitative analysis of apoptotic neurons/mm^2^ in CA3 SP of WT, Tg 3M, and Tg 6M. There are no significant differences in SP of Tg 6M and Tg 3M vs. WT mice. Data reported in all graph bars are expressed as mean ± SEM.

We performed a double staining immunohistochemistry with anti Cyt C (red) and anti NeuN (green) antibodies on hippocampal sections of TgCRND8 at 3 (Tg 3M) and 6 months of age (Tg 6M) and of WT control mice. Images of fluorescent immunostaining were taken in CA1 (Figures 7A–C,A1–C1) and CA3 hippocampal regions (Figures 7F–H,F1–H1) with epifluorescence microscopy. Figures 7D–D2 shows a Cyt C-positive neuron (arrow) in CA1 SP at a higher magnification, acquired with laser confocal microscopy.

We performed the quantitative analyses of apoptotic neurons in CA1 and CA3 SP. We found a statistically significant increase of density of apoptotic neurons in CA1 of Tg mice at 3 (+129%) and 6 months (+82%) of age in comparison to WT mice (Figure 7E) [one-way ANOVA: *F*(2,13) = 9.956, *P* = 0.0024; Newman–Keuls post-test: ^∗∗^*P* < 0.01 Tg 3M vs. WT, ^∗^*P* < 0.05 Tg 6M vs. WT; WT: *n* = 4; Tg 3M: *n* = 6; Tg 6M: *n* = 6].

The analysis of Cyt C-positive neurons performed in CA3 SP showed a slight, not statistically significant increase at 6 months of age (+36%) in comparison to WT mice, as shown by qualitative (Figures 7F–H1) and quantitative analyses (Figure 7I) [one-way ANOVA: *F*(2,12) = 0.890, *P* = 0.4362, n.s.; WT: *n* = 4; Tg 3M: *n* = 6; Tg 6M: *n* = 5].

We compared the results obtained in CA1 to those obtained in CA3 by two-way ANOVA with ROIs and experimental groups as the two variables.

Two-way ANOVA analysis demonstrated that apoptotic neurons were not significantly different in CA1 SP in comparison to CA3 SP of all groups examined (WT, Tg 3M, and Tg 6M). Nevertheless, the percent increase of apoptotic neurons in CA1 SP of Tg 3M and Tg 6M was significantly higher than in CA3 SP (see Table 1).

**Table 1:** Differences between CA1 and CA3 in all parameters investigated.

	Tg 3M		Tg 6M	
		
	CA1	CA3	CA1	CA3
Density of astrocytes in SP	+43	+8	+65^∗∗^	+5
Intensity of GFAP in SR	+33	+13	+32	+8
Length of astrocyte branches in SR	+17^∗∗^	-3	+32^∗∗^	-5
Density of IBA1^+^ microglia in SP	+7	+2	+35	+46
Density of IBA1^+^ microglia in SR	+1	-2	+72	+48
Density of CD68^+^ microglia in SP	+82	+252	+347^∗^	+1,448
Density of CD68^+^ microglia in SR	+36	+940	+902^∗^	+3,395
Volume of CD68^+^ microglia in SR	+487^∗^	+170	+626	+434
Density of triads in SR	+219	+111	+674^∗∗^	+170
Density of TNF-α^+^ cells in SR	+187	+254	+410	+1646
iNOS	+50.75	+58.86	+65.87^∗^	+1.342
IL1β	+25.11	+28.38	+83.55	+122.5
Density of Cyt C^+^ neurons in SP	+129^∗∗∗^	+10	+82	+36
Density of neurons in SP	-15^∗∗^	+7	-28^∗∗^	-8
Volume of neurons in SP	-36^∗∗∗^	+5	-42^∗∗∗^	+8
Thickness of SP	-12^∗∗^	+19	-40^∗^	+3


*All data represent percent differences from WT, taken as 100%. ^∗^*P* < 0.05; ^∗∗^*P* < 0.01; ^∗∗∗^*P* < 0.001 in comparison to CA3 (Student’s *t*-test).*

### Analysis of Neuron–Astrocytes–Microglia Triad in CA1 and CA3 Hippocampus of TgCRND8 Mice

**FIGURE 8:**
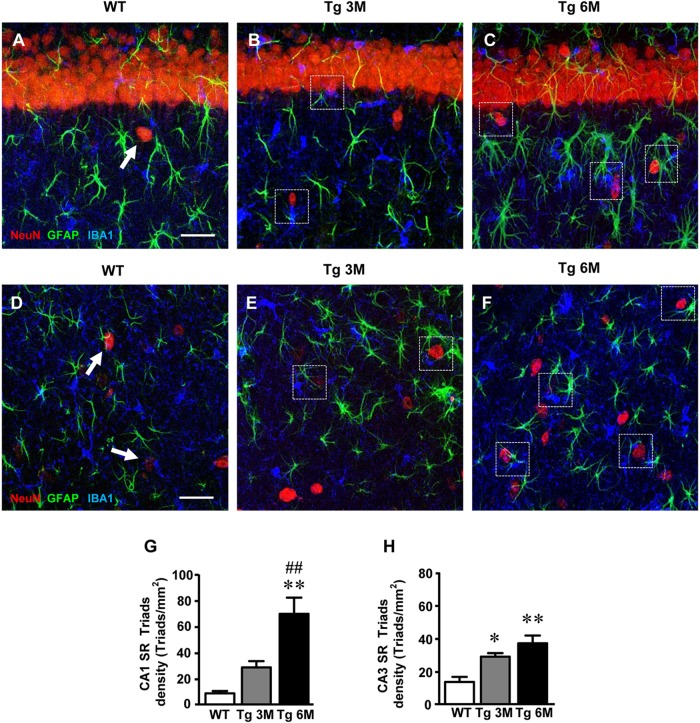
Characterization and quantification of the neuron-astrocyte-microglia triads in CA1 and CA3 SR of WT, TG 3M, and TG 6M mice. **(A–C)** Representative confocal photomicrographs of triple immunostaining of neurons (NeuN, red), astrocytes (GFAP, green), and microglia (IBA1, blue) in the CA1 SR of a WT **(A)**, a TG 3M **(B)**, and of a TG 6M mouse **(C)**. Scale bar: 40 μm. **(D–F)** Representative confocal photomicrographs of triple immunostaining of neurons (NeuN, red), astrocytes (GFAP, green), and microglia (IBA1, blue) in the CA3 SR of a WT **(D)**, a TG 3M **(E)**, and of a TG 6M mouse **(F)**. Scale bar: 40 μm. Examples of triads in Tg 3M and Tg 6M are shown in the framed areas **(B,C,E,F)**. In WT mice **(A–D)**, groups of neuron-astrocyte-microglia not considered as triads are pointed by arrows. **(G,H)** Quantitative analysis of neuron-astrocyte-microglia triads/mm^2^ in CA1 **(G)** and CA3 **(H)** SR. Triads are significantly more numerous in CA1 SR of Tg 6M vs. WT mice. In CA3 SR, triads are significantly more numerous in Tg 3M and Tg 6M than in WT mice. Data reported in all graph bars are expressed as mean ± SEM.

To evaluate the presence of neuron–astrocytes–microglia triads in CA1 and CA3 SR, we performed triple staining immunohistochemistry with anti-NeuN, anti-GFAP, and anti-IBA1 antibodies on hippocampal sections of TgCRND8 (Tg 3M, Tg 6M) and control mice (WT). Images of fluorescent immunostaining were taken in CA1 (Figures 8A–C) and CA3 (Figures 8D–F) hippocampal regions with confocal microscopy and the quantitative analysis of the density of the triads was performed in CA1 and CA3 SR. The presence of triads in CA1 and CA3 SR is shown qualitatively in Figures 8A–C and D,E, respectively.

The Quantitative analysis of triads in CA1 SR is shown in the graph in Figure 8G. We found a statistically significant increase of triads density in CA1 SR of Tg 6M (+674%) in comparison with WT mice [one-way ANOVA: *F*(2,8) = 12.66, *P* = 0.0033; Newman–Keuls post-test: ^∗∗^*P* < 0.01 Tg 6M vs. WT, ^##^*P* < 0.01 Tg 6M vs. Tg 3M; WT: *n* = 3; Tg 3M: *n* = 4; Tg 6M: *n* = 4].

In CA3 SR, we found a statistically significant increase of triads density both in Tg 3M (+111%) and Tg 6M (+170%) in comparison to WT mice (Figure 8H) [one-way ANOVA: *F*(2,9) = 11.55, *P* = 0.0033; Newman–Keuls post-test: ^∗^*P* < 0.05 Tg 3M vs. WT, ^∗∗^*P* < 0.01 Tg 6M vs. WT; WT: *n* = 4; Tg 3M: *n* = 4; Tg 6M: *n* = 4].

We compared the results obtained in CA1 to those obtained in CA3 by two-way ANOVA with ROIs and experimental groups as the two variables.

The statistical analysis on the density of triads revealed that in WT and Tg 3M animals, there was no significant difference between CA1 and CA3 SR while a significant increase was found in CA1 SR of Tg 6M. Indeed, we found a significant main effect for experimental groups [*F*(2,17) = 21.97, *P* < 0.001], and for Interaction [*F*(2,17) = 5.134, *P* < 0.05], but not for ROIs [*F*(1,17) = 3.148; n.s.]. Bonferroni post test showed that the density of triads in CA1 SR of Tg 6M was significantly higher than in CA3 SR (*P* < 0.01).

### Comparisons Between CA1 and CA3

The most salient differences between CA1 and CA3 are reported in Table 1. Each column represents the percent variation, normalized to the control values found in WT mice, of each parameter investigated in transgenic mice at 3 and 6 months of age. The differences between the parameters investigated in CA1 and CA3 at 3 or 6 months of age, were then evaluated using the Student’s *t*-test. It is evident from the data in the table that astrogliosis was higher in CA1, both in terms of density of astrocytes and of length of astrocyte branches. The number of IBA1-positive microglia did not vary significantly between the two areas, while CD68^+^ microglia was more pronounced both in SP and SR of CA3. TNF-α showed a very peculiar characteristic: it was expressed by astrocytes in CA1 SR, while in CA3 it was present not only in astrocytes but also in other structures. The percent variation of iNOS was higher in CA1 SR at 3 months of age, while that of IL1β varied similarly in CA1 and CA3 SR at 3 and 6 months of age. CA1 pyramidal neurons of transgenic mice were significantly smaller and less numerous than those of WT mice, causing significant shrinkage of CA1 stratum pyramidale. On the contrary, CA3 Pyramidal neurons of transgenic mice did not change significantly, neither in terms of number and volume and did not show significant increase of apoptosis, even at 6 months of age. The decrease of CA1 pyramidal neurons possibly was caused, at least in part, by increase of apoptotic mechanisms, which indeed were significantly more pronounced in CA1 than in CA3 SP. Interestingly, neuron–astrocytes–microglia triads were more numerous in CA1 SR than in CA3 SR. Astrocytes and microglia, forming triads with neurons, help clearing apoptotic and degenerating neurons at higher degree in CA1, in comparison to CA3.

## Discussion

Aim of the present study was to investigate and compare the quantitative, temporal, and spatial modifications of the interplay between astrocytes, microglia, and neurons in CA1 and CA3 hippocampus of TgCRND8 mice, a mouse model of Aβ deposition, at 3 and 6 months of age. The comparison between these two hippocampal areas is fundamental and can help explaining the more pronounced sensitivity of CA1 pyramidal neurons to neurodegenerative insults, both in experimental animals and in humans (Mueller et al., 2010; Small et al., 2011; Bartsch et al., 2015). It is also important because of the critical, although different, role of these areas in memory processing and because of their significant functional, structural, and morphological alterations in AD (Bartsch and Wulff, 2015). Therefore, in this work, we studied the different patterns of neuron degeneration and apoptosis, glia activation/modification, as well as different expression of proinflammatory mediators, at different stages of plaque deposition, subdividing the hippocampus in the two main ROIs CA1 and CA3. The principal findings of this study were that the two contiguous and interconnected hippocampal regions of transgenic mice display remarkably different cellular modifications and neuronal vulnerability to the deposition of Aβ plaques. Interestingly, while Aβ load in CA1 was not significantly different from that of CA3, both at 3 and 6 months of age, in CA1 SR of Tg 6M mice the Medium and Large plaques were significantly more numerous than in CA3 SR. As expected, we found many hypertrophic astrocytes surrounding and infiltrating Aβ plaques, both in CA1 and CA3. Nevertheless, astrogliosis was also evident not only close to the plaques, but also in the SR parenchyma far from and devoid of plaques. Astrogliosis, evidenced by increased recruitment of astrocytes, increased expression of GFAP and elongation of astrocyte branches, significantly increased in the entire CA1 of transgenic mice, mainly at 6 months of age, while in CA3 it was significantly lower than in CA1. Healthy astrocytes are indispensable for synaptogenesis, synaptic maintenance and maturation (Pfrieger, 2009; Heneka et al., 2010), significantly contributing to memory-associated processes (Verkhratsky et al., 2011), but they are also involved in AD, as first suggested by Alois Alzheimer (Alzheimer, 1910). The involvement of astrocytes in AD progression is variegated, depending upon the brain area interested and the gravity of the disease. Indeed, in the hippocampus, progression of AD has been associated with an early atrophy of astrocytes, defined clasmatodendrosis (Penfield, 1928; Hulse et al., 2001) that, at later stages of the disease, coexists with reactive astrocytes around plaques (Olabarria et al., 2010; Yeh et al., 2011). We never found clasmatodendrotic astrocytes in CA1 and CA3 parenchyma of transgenic mice at both 3 and 6 months of age.

Dissecting the hippocampus in the two main regions CA1 and CA3 we observed significant differences in most of the parameters investigated, at different stages of plaque deposition. In CA1 SR of 6 months old transgenic mice, we found significantly higher levels of the expression of the cytokines TNF-α, IL1β, as well as iNOS in astrocytes, confirming our and others’ results in TgCNRD8 mice (Luccarini et al., 2012) and other models of neurodegenerative diseases (Deng et al., 2014; Lana et al., 2017). It has been demonstrated that the inflammatory cytokine TNF-α is usually expressed in the brain by activated microglia, and, to a lesser extent, by activated astrocytes and neurons (Perry et al., 2002). Nevertheless, many studies have shown that the gradual deposition of Aβ peptide and overproduction of inflammatory mediators activate astrocytes, further inducing expression and release of cytokines, interleukins, NO, and other proinflammatory mediators (Choi et al., 2014; Heneka et al., 2015) that in turn increase pro-apoptotic cascades in the surrounding brain areas (Luccarini et al., 2012), and exacerbate AD pathology (Tan et al., 2007). Furthermore, it has been demonstrated that TNF-α induces apoptosis primarily through the activation of cell-surface TNF-α type I receptors that contain death domains (Park et al., 2007). In line with these data, our results demonstrate that in CA1 SP, both at 3 and 6 months of age, a significant number of pyramidal neurons underwent apoptosis, possibly targeted by increased TNF-α expression and release by astrocytes. In addition, stimulation of iNOS by cytokines in astrocytes may cause increased concentrations of NO that can be toxic to neurons. Indeed, iNOS is upregulated AD patients’ brain (Vodovotz et al., 1996) and KO of iNOS is protective in mouse models of AD (Nathan et al., 2005). These mechanisms may be the cause of the significant loss of CA1 pyramidal neurons and shrinkage of CA1 stratum pyramidale, evidenced in CA1 but not in CA3.

It is known that Aβ plaques induce production and release of pro-inflammatory cytokines by neurons and astrocytes (Hickman et al., 2008; Daria et al., 2017) which, in turn, promote a shift in microglia activity, from surveillance/maintenance mode, to execution of immune tasks. Microglia can assume two different phenotypic forms, M1 and M2 (Loane and Kumar, 2016). While M1 microglia can express and release proinflammatory cytokines (de Bilbao et al., 2009; Protti et al., 2013), M2 microglia is more active in the surveillance/maintenance of tissue homeostasis, phagocytosing apoptotic or degenerating neurons, preventing secondary inflammatory mechanisms and promoting tissue regeneration (Hu et al., 2012; Suenaga et al., 2015). In both CA1 and CA3 of transgenic mice, we found an increase of total microglia, and of reactive microglia, corresponding to the M1 phenotype, which significantly increased in CA1 SP and SR at 3 and 6 months of age. These data indicate that the inflammatory milieu triggered by plaque deposition caused increased recruitment of microglia cells, and significantly increased its reactivity. Furthermore, in agreement with data obtained by Bolmont et al. (2008) in the cortex of APPPS1 transgenic mice, we found significant spatial orientation of microglia toward Large plaques, both in CA1 and CA3 SR of Tg 6M mice.

The reactivity state of microglia in CA1 SR, together with increased astrogliosis, caused increased formation of neuron-astrocytes-microglia triads. The concerted actions of astrocytes and microglia in the formation of triads with neurons can recognize “danger signals,” including cellular debris produced from apoptotic or necrotic cells (Milligan and Watkins, 2009), and can clear the damaged neurons or neuronal debris by phagocytosis (Cerbai et al., 2012; Lana et al., 2014). Indeed, we found several reactive microglia cells that cooperated with astrocytes in the phagocytosis of degenerated neurons, mainly in SR of transgenic mice. Under physiological conditions, the effects of astrocytes and microglia are protective, removing entire neurons by phagoptosis (Lana et al., 2017), clearing dysfunctional synapses and controlling inflammation and the diffusion of cellular damage to neighboring tissue. Also, activated microglia contribute to Aβ clearance and removal of cytotoxic debris from the nervous tissue. However, phagocytosis of living healthy neurons by microglial in inflamed CNS has also been reported (Neher et al., 2012; Vilalta and Brown, 2017). Furthermore, uninterrupted microglia activation may exacerbate inflammation, increase Aβ deposition and intensify neurodegeneration (Michaud and Rivest, 2015). Indeed, in AD, as for astrocytes, pro-inflammatory and detrimental, or anti-inflammatory and even protective properties have been attributed to microglia (Mandrekar-Colucci and Landreth, 2010; Heneka et al., 2015; Heppner et al., 2015). All these different findings may suggest that microglia may acquire heterogeneous activation states, or, as in our results, microglia can be protective or detrimental, depending on the region where it is located and other concauses.

Here, we have demonstrated that not only the proximity to plaques determines the development of a specific reactive phenotype of microglia (Plescher et al., 2018) and astrocytes (Olabarria et al., 2010), but also the different plaque size and distribution within different brain areas. In CA1 and CA3 hippocampus, although Aβ load was similar, plaque organization in terms of dimensions was different, and glia and neurons responded with differential patterns of activation and neurodegeneration. The sensitivity of the subregional pyramidal neurons to neurodegeneration was very different, at both 3 and 6 months of age. Our paper is in line with recent evidence showing hippocampal subregional-specific patterns of neurodegeneration at different stages of Aβ deposition (Albuquerque et al., 2015; Mahar et al., 2017).

Significant progress has been made in understanding the relationships of amyloid pathology to hippocampal dysfunctions; however, complete understanding of this process across hippocampal anatomical areas remains incomplete. Although the hippocampus is often described as a unitary structure, this is hardly the case. The unique molecular and synaptic milieu of its spatial domains allow asking how AD pathophysiology can arise in one region vs. the other one. Memory impairment, particularly episodic and spatial memory, is the most important symptom of AD, often related to the dysfunction of pyramidal neurons in CA1 and entorhinal cortex (Hyman et al., 1984; West et al., 2000; Scheff et al., 2007; Kerchner et al., 2010). Studies have shown that lesions centering on CA1 are sufficient for memory impairment (Zola-Morgan et al., 1986). Indeed, our data lend support to the idea that Aβ load exerts greater effects on CA1 than on CA3. Many authors found that the CA1 is the most vulnerable region of the hippocampus to neuronal loss both in animal models of AD and in AD patients (West et al., 2000; Price et al., 2001; Rossler et al., 2002; Stepanichev et al., 2006; Corbett et al., 2013). Among AD patients, variable degree of atrophy between CA1 and CA3 is often found, CA3 being the least damaged area (Hyman et al., 1984; West et al., 1994). On the other hand, in different transgenic mouse models of AD it has also been found (Gruart et al., 2008) that learning, and memory deficits are not directly correlated to Aβ load. Therefore, other factors, besides Aβ deposition may be involved in memory deficits.

The loss of CA1 pyramidal neurons, which underwent neuronal death by apoptosis, caused shrinkage of the CA1 pyramidal cell layer. All these modifications may be at the basis of memory loss which was repeatedly demonstrated in this transgenic mouse model of Aβ deposition, even at early stages (Chishti et al., 2001; Hamm et al., 2017). Interestingly however, it has been shown that increased physical activity improves behavioral and cognitive deficits in murine models of AD (García-Mesa et al., 2011) and reduces the risk of AD in humans (Buchman et al., 2012). The precise mechanism of action underlying the positive effects of physical activity are still not known, but may involve astrocytes (Latimer et al., 2011), indicating that astrogliosis may become a therapeutic target for AD (Colangelo et al., 2014).

## Author Contributions

MG designed the research. FU, DL, PN, DN, and DP performed the experiments. FU, DL, PN, DP, and MG analyzed the data. DL, MG, and FC interpreted the results and the experiments. DL, MG, FU prepared the figures. MG and DL drafted the manuscript. DL, MG, FU, and FC edited and revised the manuscript. All authors read and approved the final version of the manuscript.

## Conflict of Interest Statement

The authors declare that the research was conducted in the absence of any commercial or financial relationships that could be construed as a potential conflict of interest.

## Footnotes

1 http://rsb.info.nih.gov/ij
